# Integrative set enrichment testing for multiple omics platforms

**DOI:** 10.1186/1471-2105-12-459

**Published:** 2011-11-25

**Authors:** Laila M Poisson, Jeremy M Taylor, Debashis Ghosh

**Affiliations:** 1 Department of Public Health Sciences, Henry Ford Hospital, 1 Ford Place, Detroit, MI, 48202, USA; 2 Department of Biostatistics, University of Michigan, 1420 Washington Heights, Ann Arbor, MI, 48109-2029, USA; 3 Departments of Statistics and Public Health Sciences, Penn State University, 514A Wartik Laboratory, University Park, PA, 16802, USA

## Abstract

**Background:**

Enrichment testing assesses the overall evidence of differential expression behavior of the elements within a defined set. When we have measured many molecular aspects, e.g. gene expression, metabolites, proteins, it is desirable to assess their differential tendencies jointly across platforms using an integrated set enrichment test. In this work we explore the properties of several methods for performing a combined enrichment test using gene expression and metabolomics as the motivating platforms.

**Results:**

Using two simulation models we explored the properties of several enrichment methods including two novel methods: the logistic regression 2-degree of freedom Wald test and the 2-dimensional permutation p-value for the sum-of-squared statistics test. In relation to their univariate counterparts we find that the joint tests can improve our ability to detect results that are marginal univariately. We also find that joint tests improve the ranking of associated pathways compared to their univariate counterparts. However, there is a risk of Type I error inflation with some methods and self-contained methods lose specificity when the sets are not representative of underlying association.

**Conclusions:**

In this work we show that consideration of data from multiple platforms, in conjunction with summarization via *a priori *pathway information, leads to increased power in detection of genomic associations with phenotypes.

## Background

In biomedical studies we are often interested in comparing two groups of samples on a collection of measured variables that are possibly associated with group status. When the explanatory variables of interest are measurements from a high-throughput molecular assay, thousands of comparisons may be performed. The resulting list of differential genes from a gene expression array can be unwieldy with hundreds of entries. For metabolomics the number of molecules measured is reduced by at least an order of magnitude compared to gene expression assays [1,2], but the list of differential molecules can still be lengthy with respect to the number of leads that can be feasibly followed. Given this, researchers are often interested in grouping these lists of differentially expressed molecules into sets with common functionality. The area of enrichment testing looks at an *a priori *defined set, such as from KEGG (Kyoto Encyclopedia of Genes and Genomes) or GO (Gene Ontology) [3], and asks if the number of differentially expressed elements in the set is remarkable; either more or less than expected.

Enrichment tests work by assessing the overall evidence of differential expression behavior of the elements (e.g. genes, metabolites) within the set. The pitfall with a smaller list, such as with metabolomic analysis, is that the sets of interest may not be well-represented for testing. For instance, 67% enrichment sounds impressive unless there are only 3 molecules measured. In this case it may not be statistically significant, and it is also not clear if it is biologically interesting. When we have measured many molecular aspects, e.g. gene expression, metabolites, proteins, it is reasonable to assess their differential tendencies jointly across platforms. Integration of omics technologies has been beneficial in other areas resulting in more interpretable results (e.g., [4]) and more meaningful associations [5] than when the platforms are assessed separately. In an effort to translate this success to the area of set enrichment we explore joint set enrichment tests that incorporate the multiple platforms into a single test.

We begin with an overview of enrichment testing theory and detail the methods of interest. Using simulation we explore the properties of these methods and make comparisons and recommendations. A metabolomics dataset [1] and matched gene expression dataset are used to apply the top methods to real data. For clarity, the following exposition is presented with respect to genes and metabolites, but the results should readily apply to any high-throughput molecular measures that (1) are expected to be related within the cell or tissue, (2) can be assessed for association between clinical groups, and (3) can be assigned into *a priori *sets. Further details, as well as the R code for the simulations presented here is available as additional material (Additional Files 1 and 2) so that the reader may explore specific scenarios of interest.

### Enrichment Testing

Enrichment testing methods have been classified into two general flavors; competitive and self-contained [6,7]. We briefly introduce these testing styles and highlight the pros and cons of each method. For reference we define the 2 × 2 classification depicted in Table 1. Here *g *genes have been individually tested for differential expression and an interesting set of genes *S *has been defined. We classify each of the *g *genes by whether they are differentially expressed (*D*) and whether they are in the set of interest (*S*).

**Table 1:** General schema for a pathway enrichment test

	Differential gene (*D*)	Non-differential gene (*D'*)	Total
In the set (*S*)	*g*_*SD*_	*g*_*SD'*_	*g*_*S*_
Not in the set (*S'*)	*g*_*S'D*_	*g*_*S'D'*_	*g*_*S'*_

Total	*g*_*D*_	*g*_*D'*_	*g*

The general scheme for a hypergeometric test of differential genes is based on *g *genes divided by the criteria of inclusion in the set of interest (*S*) and inclusion in the set of differential genes (D).

#### Competitive Tests

For a set of genes, *S*, a competitive test assesses whether the amount of differential expression differs from that of its complement *S'*. The competitive null hypothesis, $H_{0}^{comp}$, then assumes that genes within the set *S *show the same amount of association with the phenotype as those in set *S' *[6]. In this way each gene set *competes *against its complementary set of measured genes.

A popular competitive set enrichment test is the Fisher's Exact test run on Table 1. Independence of the columns and rows is assessed and a statistically significant result that rejects the null hypothesis implies that the rate of differentially expressed genes is associated with set status. As it is a two-sided test, a detected association may be due to enrichment or depletion of differential genes.

The chief complaint against competitive enrichment tests is the relative enrichment estimation of a set *S *can differ depending upon the gene sets used for the reference set *S' *[8]. Though viewed as a limitation, critics concede that relative estimation is useful when there is a large number of genes that are differentially expressed.

Most competive tests rely on gene-resampling to generate the null distribution [6,7]. Empirical estimates of the null hypothesis are generated by randomly sampling *g*_*S *_genes from *S *∪ *S' *and repeating the test on these randomly generated sets. Arguments against gene-resampling methods are three-fold. First, lack of independence between genes is contrary to the assumption of exchangability in gene resampling. Second, the null distribtution is based on the selection of a new gene set not a new sample set. Finally, the sample size is dependent upon the number of genes, which is often substantially larger than the number of samples. Logistic regression was introduced by [9] as an alternative test to the Fisher's Exact test which does not require dichotomization of the genes by differential ability. The model proposed is *logit*(*Pr*(*G*_*j *_∈ *S*)) = *γ*_0 _+*γx*, for $x=-log_{10}\left(p_{j}^{G}\right)$ where $p_{j}^{G}$ is the p-value from the per-gene test of differential expression for gene *j*. The test of $H_{0}^{LR}:\gamma =0$ can be obtained from standard statistical software using a 1-degree of freedom Wald test where rejection of $H_{0}^{LR}$ indicates enrichment or depletion of the set.

#### Self-contained Tests

In contrast to competitive tests, self-contained tests do not utilize *S' *in the assessment of *S*. Specifically, only the first row of Table 1 is considered. The self-contained null hypothesis, $H_{0}^{sc}$, assumes that the gene set *S *does not contain any genes whose expression levels are associated with the phenotype of interest [6]. A binomial test of proportions based on *g_SD _*~ *binomial*(*g*_*S*_, *α*), where *α *is an expected rate of differential genes (e.g., *α *= 0.05) is an example of a self-contained test. Since only the set of interest *S *is considered a self contained test is not relative. Thus it reduces to a test of differential expression for a single gene and expands to a global test of differential expression when the entire array is the set of interest. Arguments against self-contained tests focus on the strong null hypothesis in relation to its biological interpretation wherein a single differentially expressed gene may be able to give enough evidence to reject the null hypothesis.

Self-contained tests primarily utilize subject-resampling methods to determine the null distribution of the test statistic [7]. Subject-resampling assumes that the subjects are independent and that under the null hypothesis the sample labels are randomly assigned. In contrast to gene-resampling, subject-resampling (1) follows the experimental design of most studies by assuming that the subjects are independent realizations of the study population, (2) retains the between-gene correlation structure, (3) is reflective of the number of subjects, and (4) provides a p-value that is generalizable to experiments with new subjects under study. However, subject-resampling tests are limited by their small sample size and the null hypothesis being tested by subject sampling may be difficult to state.

An example of a self-contained test that utilizes the strength of differential expression per element without dichotomization is the sum of squared test statistics. Begining with the per-element test statistics of differential ability, ${\underset{-}{T}}^{G}=\left(T_{1}^{G},T_{2}^{G},\ldots T_{g}^{G}\right)$, we simply sum all squared test statistics within set *S*, $W_{S}^{G}=\sum_{j\in S}{\left(T_{j}^{G}\right)}^{2}$ for *j *= 1,..., *g *genes [10]. Significance of $W_{S}^{G}$ is determined by generating the null distribution using permutation of sample labels to form null datasets.

## Methods

### Approach: Joint Assessment of Enrichment

In this work we are interested in tests of enrichment that incorporate both the gene expression and metabolite information. We begin with per-gene and per-metabolite tests of differential expression. We define two vectors of test statistics, specifically two-sample t-tests, ${\underset{-}{T}}^{G}=\left(T_{1}^{G},T_{2}^{G},\ldots ,T_{g}^{G}\right)$ and ${\underset{-}{T}}^{M}=\left(T_{1}^{M},T_{2}^{M},\ldots ,T_{m}^{M}\right)$, and two corresponding vectors of p-values, ${\underset{-}{P}}^{G}=\left(P_{1}^{G},P_{2}^{G},\ldots ,P_{g}^{G}\right)$ and ${\underset{-}{P}}^{M}=\left(P_{1}^{M},P_{2}^{M},\ldots ,P_{m}^{M}\right)$, for *g *genes and *m *metabolites, respectively.

#### Concatenation of Lists

Univariate tests can be employed directly as a means of joint assessment by concatenating the per-gene and per-metabolite test statistics to form a single vector of data, e.g. $\underset{-}{T}=\left({\underset{-}{T}}^{G},{\underset{-}{T}}^{M}\right)$. Care must be taken that the joined vectors are made comparable before concatenation. For instance, two-sample t-test statistics are not comparable if they are from different sample sizes as they have different degrees of freedom. P-values, being comparable by design, will resolve this problem, however, they lose directionality, which may be of interest, and empirical p-values may lack precision. Additionally, concatenation of the lists may lead to bias favoring the larger dataset [11].

#### P-value multiplication

To arrive at a single statistic from two tests we can use a p-value combining method such as Fisher's method [12]. Here we assume that $-2\times \left(log_{e}P_{S}^{M}+log_{e}P_{S}^{G}\right)\sim X_{4}^{2}$ for the enrichment p-values for metabolites and genes in set *S*. The $X^{2}$ distribution is not technically correct when the tests being summed are dependent, which is likely the case here. However, since this is a commonly used method we wished to incude it in the comparison.

#### Logistic regression analysis with 2-df Wald test

We propose a multivariate extension of the competitive logistic regression test of [9]. First the genes and metabolites are modelled separately using the absolute value of the per-element t-statistic as the measure of differential ability. Thus for set *S *we fit models (1) $logit\left(Pr\left(G_{j}\in S\right)\right)=\gamma_{0}+\gamma |T_{j}^{G}|$ and (2) $logit\left(Pr\left(M_{k}\in S\right)\right)=\mu_{0}+\mu |T_{k}^{M}|$. (We use the absolute t-statistic, instead of -*log*_10_(*p*), because it is more stable in the bootstrap resampling described below.)

We next construct a joint test of $H_{02}^{LR}:\gamma =0,\mu =0$ using a two degree-of-freedom Wald test, *EV*^-1^*E*^*T*^, where $E=\left[\hat{\gamma },\hat{\mu }\right]$, and *V *is an estimated variance-covariance matrix for *γ *and *μ*. Estimates of the variance of *γ *and *μ *are available from the univariate models but the covariance term is not easily obtained. We attempted to estimate this covariance measure through bootstrap simulation but we find that there is near-zero correlation between the parameters $\hat{\gamma }$ and $\hat{\mu }$ even in sets where the genes and metabolites were simulated to be correlated. The loss of correlation is due to row-resampling used for this bootstrap estimation, however, subject sampling severly underestimates the parameter variance compared to the variance estimates obtained from the univariate models. For more discussion of this bootstrap estimation, we refer the reader to Additional File 1.

Given this we estimate *V *as a diagonal matrix with $\sigma_{\gamma }^{2}=var\left(\hat{\gamma }\right)$ and ${\sigma_{\mu }}^{2}=var\left(\hat{\mu }\right)$ obtained from the univarite models. This reduces our test statistic to the sum of the two one-degree-of-freedom tests which can be written as $U_{S}^{LR}=EV^{-1}E^{T}={\hat{\gamma }}^{2}\sigma_{\gamma }^{-2}+{\hat{\mu }}^{2}\sigma_{\mu }^{-2}$, for set *S*, where $E=\left[\hat{\gamma },\hat{\mu }\right]$, and $V=diag\left(\sigma_{\gamma }^{2},\sigma_{\mu }^{2}\right)$. We assume that $U_{S}^{LR}\sim X_{2}^{2}$ under the null hypothesis $H_{02}^{LR}:\gamma =0,\mu =0$.

#### Sum of squared statistics with 2-dimensional permutation test

We propose a multivariate extension of the self-contained sum of squared statistics [10]. First, we obtain the enrichment test statistic for each of the metabolites and the genes in set *S*, $\left(W_{S}^{G},W_{S}^{M}\right)$. Next, we assess this observed statistic pair against the distribution of null estimates pairs ${\left({\tilde{W}}_{S}^{G},{\tilde{W}}_{S}^{M}\right)}_{h},h=1,\ldots ,H$. Marginally, $P_{S}^{G}=H^{-1}\sum_{h=1}^{H}I\left({\left({\tilde{W}}_{S}^{G}\right)}_{h}\geq {\hat{W}}_{S}^{G}\right)$ for genes and $P_{S}^{M}=H^{-1}\sum_{h=1}^{H}I\left({\left({\tilde{W}}_{S}^{M}\right)}_{h}\geq {\hat{W}}_{S}^{M}\right)$ for metabolites. We then calculate the Mahalanobis distance from the observed statistics $\left({\hat{W}}_{S}^{G},{\hat{W}}_{S}^{M}\right)$ to the centroid of the cloud of permutation statistic pairs.

For a set of *H *null pairs $\left({\tilde{W}}_{S}^{G},{\tilde{W}}_{S}^{M}\right)$, define the centroid $\left(\psi_{H}^{G},\psi_{H}^{M}\right)$ and variance-covariance matrix *V*_*H*_. Then the Mahalanobis distance can be calculated for any pair $\left(W_{S}^{G},W_{S}^{M}\right)$ as $D_{SH}\left(W_{S}^{G},W_{S}^{M}\right)={\left({\left(\left(W_{S}^{G},W_{S}^{M}\right)-\left(\psi_{H}^{G},\psi_{H}^{M}\right)\right)}^{T}V_{H}^{-1}\left(\left(W_{S}^{G},W_{S}^{M}\right)-\left(\psi_{H}^{G},\psi_{H}^{M}\right)\right)\right)}^{1∕2}$ including the observed pair $\left({\hat{W}}_{S}^{G},{\hat{W}}_{S}^{M}\right)$[13]. Thus to calculate the joint permutation p-value for $\left({\hat{W}}_{S}^{G},{\hat{W}}_{S}^{M}\right)$ we calculate

$$P_{S}^{GM}=\frac{1}{H}\overset{H}{\underset{h=1}{\sum }}I\left(D_{SH}{\left({\tilde{W}}_{S}^{G},{\tilde{W}}_{S}^{M}\right)}_{h}\geq D_{SH}\left({\hat{W}}_{S}^{G},{\hat{W}}_{S}^{M}\right)\right)$$

**Figure 1:**
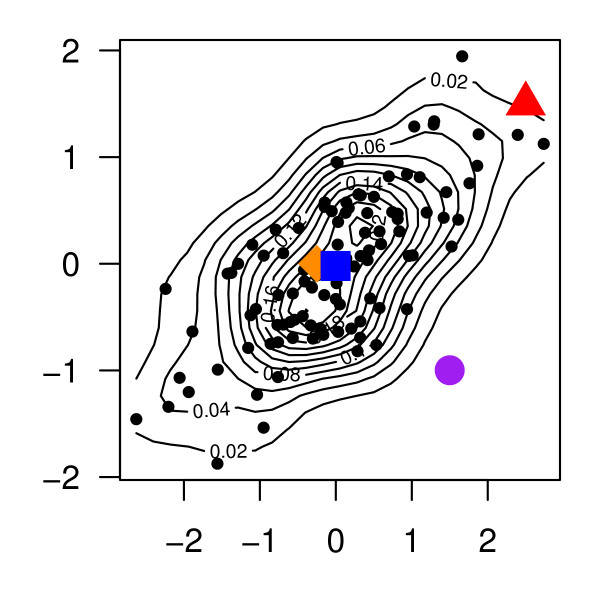
**Mahalanobis distance plot example**. A contour plot overlaying the scatterplot of 100 random draws from a bivariate normal distribution with mean zero, unit variance, and 50% correlation. The centroid defined by the marginal means is noted by a blue square. Three points of interest are added as the orange diamond, red triangle, and purple circle. If any of these points had been the observed value that gave rise to this null distribution its p-value would be 0.94, 0.04, < 0.01, respectively.

We chose the Mahalanobis distance metric since it accounts for the shape or spread of the null distribution [13]. The utility of this is apparent in Figure 1, where a hypothetical cloud of points is drawn as the null distribution. We consider 3 points that could have given rise to this null cloud. The orange diamond, being near to the centroid (blue square) is surpassed by most null values resulting in a p-value of 0.94. The red triangle is on the edge of the null distribution but is still surpassed by four null points so it is given a p-value of 0.04. The purple circle is outside of the cloud of null points and thus results in a p-value < 1/*H*. Points outside the null cloud are of most interest because they would be missed marginally. Additionally, had we not accounted for the non-spherical shape of the null distribution (e.g., using an Euclidean distance measure) the purple circle would not have been identified as extreme.

### Simulation Models

We use simulation to assess the properties of the joint enrichment tests described above. The two simulation models used are presented in the following. Further details and simulation code are available online as additional material (Additional Files 1 and 2).

#### Simulation I: Disjoint Set Simulation

In this simulation model we assume that the genes and metabolites can be separated into fifty equally-sized disjoint sets. That is each gene and metabolite is included in only one set. The correlation structure is the same for each set but no correlation is assumed between sets. Additionally, ten sets are simulated to have association with disease and the level of enrichment is consistent across these sets. This simple model with homogeneous sets allows us to explore specific hypotheses about the properties of the methods.

Define *Y*_*ij *_as the gene expression measurement for sample *i *and gene *j*. Likewise let *Z*_*i'k *_be the metabolite intensity measure for sample *i' *and metabolite *k*. The gene expression measures and metabolite measures need not arise from the same subjects, but it is possible for some or all samples to be matched by subject, i.e. *i *= *i'*. Define *Q*_*i *_and *Q*_*i*' _as the case-control status for samples *i *and *i'*, respectively, where they take values 1 for case and 0 for control.

We divide the data, *Y *and *Z*, into *s *sets. Let *I*_*S *_be an indicator for association of set *S *with case status, *I*_*S *_= 1 if the set is associated with phenotype and 0, otherwise. Here and in the sequel, *S *will be the index for gene set, and *s *will represent the total number of sets. Furthermore, define the indicator variables *g*_*jS *_and *m*_*kS *_for the inclusion of gene *j *and metabolite *k*, respectively, in set *S*, such that

$$g_{jS}=\left\{\begin{array}{cc} 1, & \text{Gene}\;j\;\text{in}\;\text{set}\;S\\ 0, & \text{otherwise} \end{array}\right)$$

and

$$m_{kS}=\left\{\begin{array}{cc} 1, & \text{Metabolite}\;k\;\text{in}\;\text{set}\;S\\ 0, & \text{otherwise} \end{array}\right)$$

Define *D*(*I*_*S*_*g*_*jS*_)_*j *_and *C*(*I*_*S*_*m*_*kS*_)_*k *_to be indicator variables of differential expression between cases and controls for genes and metabolites, respectively. We use set association to define Bernoulli distributions for *D*(*I*_*S*_*g*_*jS*_)_*j *_and *C*(*I*_*S*_*m*_*kS*_)_*k *_such that a gene or metabolite has probability *d*_1 _or *c*_1 _of being differentially expressed provided that it is in at least one associated set. In other words,

$$D{\left(I_{S}g_{jS}\right)}_{j}\sim \{\begin{array}{c} Bern(d_{1}),\text{if}\;max_{S}(I_{S}g_{jS})=1;\\ Bern(d_{0}),\text{otherwise} \end{array}$$

and

$$C{\left(I_{s}m_{ks}\right)}_{k}\sim \{\begin{array}{c} Bern(c_{1}),\text{if}\;max_{S}(I_{S}m_{kS})=1\\ Bern(c_{0}),\text{otherwise} \end{array}$$

Here *d*_1_, *d*_0_, *c*_1_, and *c*_0 _are fixed values that can be set in the simulation. To simulate set enrichment we assign *d*_1 _>*d*_0 _and *c*_1 _>*c*_0_. Interestingly, as *d*_1 _→ *d*_0 _(or as *c*_1 _→ *c*_0_) the effect of being in the set diminishes under the competitive definition of enrichment. However, tests of the self-contained null hypothesis will not be affected provided that *d*_1 _and *c*_1 _are still sufficiently large.

Let us then write the simulation model as:

(1) $$\begin{array}{c} Y_{ij}=\alpha +\beta_{j}+\omega_{j}D{\left(I_{S}g_{jS}\right)}_{j}Q_{i}+e_{Y_{ij}}\\ Z_{i^{\prime }k}=\theta +\phi_{k}+\eta_{k}C{\left(I_{S}m_{kS}\right)}_{k}Q_{i^{\prime }}+e_{Z_{i^{\prime }k}} \end{array}.$$

This additive model allows for a non-zero global mean expression (intensity) level through *α *(*θ*). It assumes a mean expression (intensity) level per gene (metabolite) as defined by *β*_*j *_(*φ*_*k*_), which is modified for case samples by *ω_j _*(*η*_*k*_) according to the distributions *D*(*I*_*S*_*g*_*jS*_)_*j *_(*C*(*I*_*S*_*m*_*kS*_)_*k*_). We allow $\rho_{MG}=Corr\left(e_{Y_{ij}},e_{Z_{i^{\prime }k}}\right)>0$ in order to simulate matched samples (i.e. *i *= *i*'). We also allow correlation between genes $\rho_{GG}=Corr\left(e_{Y_{ij}},e_{Y_{ij^{\prime }}}\right)$ and between metabolites $\rho_{MM}=Corr\left(e_{Z_{i^{\prime }k}},e_{Z_{i^{\prime }k^{\prime }}}\right)$. In this simulation model these correlations are limited to genes and metabolites within the same set, thereby reducing the complexity of the simulated data structure.

*Y*_*i *_and *Z*_*i' *_are drawn from a multivariate normal distribution, $\left({\underline{Y}}_{i}^{\prime },{\underline{Z}}_{i}^{\prime }\sim MVN((\beta ,\phi ),{\underline{\underline{\sigma }}}_{YZ}\right)$, where *β*_*j *_and *φ*_*k *_are drawn from normal distributions with zero mean and variances 4*σ*^2 ^with $\sigma^{2}\sim X_{4}^{-2}$ are drawn for each of *g *genes and *m *metabolites. The covariance matrix ${\underline{\underline{\sigma }}}_{Y\;Z}$ uses the element-wise variances used for *β*_*j *_and *φ*_*k *_for the diagonal variance entires and constant covariances defined to retain the desired between-element correlations for the off-diagonal entries. The modifiers are drawn from *ω*_*j *_~ *Unif*([-2.5, -0.5] ∪ [0.5, 2.5]) and *η*_*k *_~ *Unif*([-1.5, -0.5] U [0.5, 1.5]) and added to the multivariate normal results according to the *g*_*jS *_and *m*_*kS *_indicators. The smaller range on *η*_*k *_creates weaker intensity differences for the metabolites; this reflects our real experience with this platform and provides another dimension for comparison.

For this simulation, we assume that the number of samples is the same for cases and controls, with *N*_*sample *_∈ (30,100). We allow the correlations to vary: *ρ*_*YY *_= *ρ*_*ZZ *_∈ (0.2, 0.6) and *ρ*_*YZ *_∈ (0.10, 0.25) where *ρ*_*GG *_= *ρ*_*MM *_>*ρ*_*MG*_. We consider gene sets with 20 measurements, i.e. $N_{G_{S}}=20,\;N_{M_{S}}=4$, and metabolite sets with $N_{M_{S}}\in \left(4,20\right)$. The enrichment levels (*d*_1_, *d*_0_) and (*c*_1_, *c*_0_) are allowed to vary with (*d*_1_, *d*_0_) = (*c*_1_, *c*_0_) ∈ [(0.5,0), (0.25,0), (0.10, 0), (0.25, 0.05), (0.05,0.05), (0.10,0.10), (0, 0)] with the last three pairs representing null models for the competitive tests, the last being null for the self-contained test.

#### Simulation II: Heterogeneous Set Simulation

This simulation model generates the same number of genes and metabolites in total and per-set as for the disjoint simulation above. The simulation model of Equation 1 is used as the basis of the data generation. However, *m*_*kS*_, *g*_*jS*_, *D*_*j*_, and *C*_*k *_are fixed to construct clusters of genes and metabolites with varying levels of association with disease. We also allow the sets to overlap and to be non-homogeneous in correlation structure and enrichment. This style of simulation was used by [10] in their review of various single-platform enrichment tests. Here we can assess how well the methods are able to detect various set types in a non-homogeneous setting. Null sets are also included providing a reference for competitive tests and to allow estimation of false discovery rates.

The *N*_*gene *_× *N*_*sample *_gene expression matrix and *N*_*metabolite *_× *N*_*sample *_metabolite intensity matrix are drawn in blocks of $N_{G_{s}}\times N_{sample}$ genes and $N_{M_{s}}\times N_{sample}$ metabolites according to multivariate normal distributions *h *= 1,..., 9; see Table 2. These nine distributions have varying levels of correlation and enrichment between genes and metabolites. The remaining genes and metabolites required to reach size *N*_*gene *_and *N*_*metabolite*_, respectively, are drawn from distribution *h *= 0 to represent the null elements. As in the disjoint simulation, we set $N_{G_{s}}=20$ and $N_{M_{s}}$ to be either 4 or 20. This results in 1000 genes and either 200 or 1000 metabolites. The data are correlated for some genes and some metabolites, though not all are correlated, see Table 3. The overall rate of differential expression is 12% for the genes and 12% for the metabolites in each dataset. To assess the enrichment tests on a variety of set structures we subset the *N*_*gene *_× *N*_*sample *_and *N*_*metabolite *_× *N*_*sample *_data matrices into 70 sets. The first 24 sets, described in Table 3, show various levels of enrichment. The next five sets (25-29) are determined by a random draw from all simulated genes and metabolites. These random sets assess the rate of non-specific set identification since genes and metabolites are selected across all distributions *h *= (0,1,..., 9). Finally, the remaining 41 sets (30-70) are a partitioning of the null elements, *h *= 0, so that each element participates in at least one set. The null sets allow us to assess false discovery error rates. The set participation indicators, *g*_*jS *_and *m*_*kS *_for set *S*, gene *j *and metabolite *k*, respectively, are determined at the start of the analysis. Random draws as required for sets 10 - 29 are done once fixing set membership throughout the analysis as we would expect in non-simulated data. For clarity, an example of the indicator matrix, *m*_*kS*_, for the inclusion of metabolite *k *in set *S*, is given in Additional File 1.

**Table 2:** Structure of the multivariate distributions in the disjoint pathway simulation

Distribution (h)	0	1	2	3	4	5	6	7	8	9
% Diff. Genes	0	100	100	100	100	100	100	0	0	0
% Diff. Metab.	0	100	100	100	0	0	0	100	100	100
*ρ*_*YY*_, *ρ*_*ZZ*_	0	0	*r*_1_	*r*_1_	0	*r*_1_	*r*_1_	0	*r*_1_	*r*_1_
*ρ*_*YZ*_	0	0	0	*r*_2_	0	0	*r*_2_	0	0	*r*_2_

The data are generated from 10 multivariate distributions (see Equation 1) with the following correlation structures and differential patterns.Twenty genes and four metabolites are drawn from each distribution (*h *= 1,...,9). Background genes (n = 820) and metabolites (m = 164) are simulated to be non-differential and without correlation, see distribution *h *= 0. In this work we consider *r*_1 _∈ (0.2, 0.6) and *r*_2 _∈ (0.1, 0.25) provided that *r*_1 _>*r*_2_.

**Table 3:** Set definitions for the disjoint pathway simulation

*π*	h	1	2	3	4	5	6	7	8	9
1		1	2	3	4	5	6	7	8	9
0.5		10	11	12	13	14	15	16	17	18
0.25		19	20	21	22	23	24	-	-	-

Each set *s*, *s *= 1,..., 24, contains $N_{G_{s}}$ genes and $N_{M_{s}}$ metabolites drawn such that with probability *π *the elements are from a differential distribution *h *∈ (1, 2..., 9) and the remainder are from the null distribution *h *= 0.

Using the simulation models above we explored the behavior the Fisher's exact test, logistic regression, and sum of squared statistics test when applied jointly to two data sets. We combined the data via concatenation, the Fisher's method of combining p-values, and the multivariate extensions of logistic regression and the sum of squared statistics.

## Results and Discussion

### Heterogeneous set simulation results

**Figure 2:**
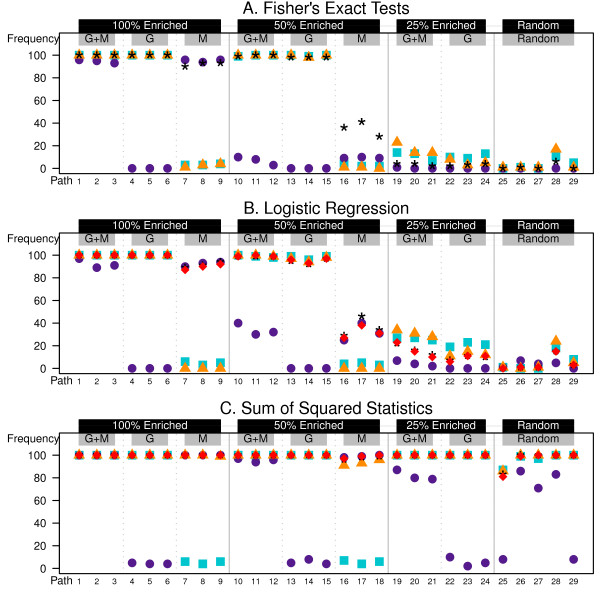
**Heterogeneous simulation results $\left(N_{G_{s}}=N_{M_{s}}=20\right)$**. 1000 genes and 1000 metabolites generated for 30 samples. Genes (G), Metabolites (M), or both (G + M) are differentially expressed within the set with probability according to the enrichment percentage listed in the black boxes at the top of the plot. Each trio of columns, from left to right, represents no correlation (*ρ*_*GG *_= *ρ*_*MM *_= *ρ*_*MG *_= 0), correlation within element only (*ρ*_*GG *_= *ρ*_*MM *_= 0.20, and *ρ*_*MG *_= 0), and correlation within and between elements (*ρ*_*GG *_= *ρ*_*MM *_= 0.20, and *ρ*_*MG *_= 0.10). Sets 25,..., 29 were chosen randomly. The symbols represent the frequency of rejecting the null hypothesis in 100 simulated datasets. [Blue square, univariate gene; Purple circle, univariate metaboltie; Orange triangle, concatenation; Black star, Fisher's method; Red diamond, multivariate extension]

Let us first consider some results from the heterogeneous set simulations which provide an overview of the behavior of the methods. For each simulation scenario, 100 datasets were generated and tested. In Figure 2 we depict the frequency with each set, from 1 - 29, was determined to be significant at *α *= 0.05 for each test considered. The average rate of false positives is computed across the 41 null sets per test.

Looking at the univariate tests in Figure 2 (univariate gene, blue square; univariate metabolite, purple circle) we see the behavior that we would expect for the different test styles. The tests are able to detect sets 1-9 according to their enrichment. Genes but not metabolites are detected for sets 4-6. Metabolites but not genes are detected for sets 7-9 but not perfectly in the competitive Fisher's exact and logistic regression tests (panels A and B), due to the small set size, $N_{M_{s}}=4$. The Fisher's exact test loses all ability to detect metabolite enrichment beyond 100% enrichment. The logistic regression model has about 50% detection of the metabolite sets when they are 50% enriched, sets 10-12 and 16-18, highlighting the power gain with non-dichotomized tests. The self-contained sum-of-squared statistic (panel C) test only begins to have trouble detecting the metabolite enrichment at 25% enrichment (sets 19 - 21).

When we turn our attention to the multivariate methods (red diamonds) we see a similar pattern to the results from p-values combined by Fisher's method (black stars). For the competitive tests these methods tend to follow the gene expression data results. There is some improvement in the metabolite only sets (i.e., sets 16-18). For the logistic regression test they also show a moderate effect, between that of the gene and metabolite only tests for sets 19-24 (panel B). All methods perform maximally in the self-contained sum-of-squared statistics test and increased power is provided to sets 19-21 (panel C).

**Figure 3:**
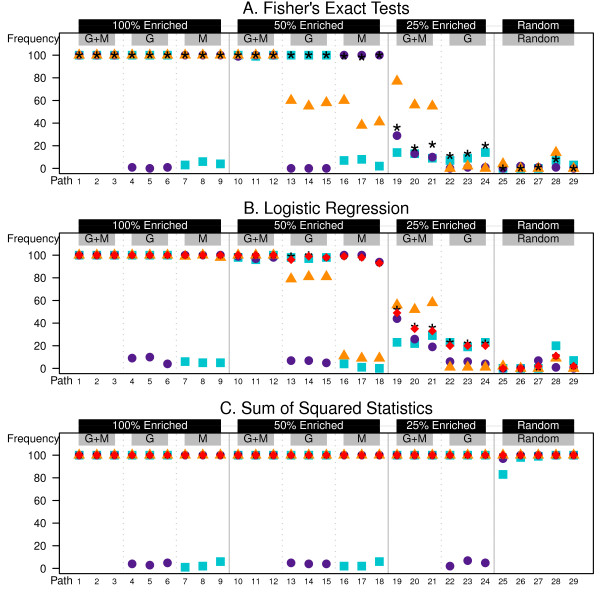
**Heterogeneous simulation results ($N_{G_{S}}=20,\;N_{M_{S}}=4$)**. 1000 genes and 200 metabolites generated for 30 samples. Genes (G), Metabolites (M), or both (G + M) are differentially expressed within the set with probability according to the enrichment percentage listed in the black boxes at the top of the plot. Each trio of columns, from left to right, represents no correlation (*ρ*_*GG *_= *ρ*_*MM *_= *ρ*_*MG *_= 0), correlation within element only (*ρ*_*GG *_= *ρ*_*MM *_= 0.20, and *ρ*_*MG *_= 0), and correlation within and between elements (*ρ*_*GG *_= *ρ*_*MM *_= 0.20, and *ρ*_*MG *_= 0.10). Sets 25,..., 29 were chosen randomly. The symbols represent the frequency of rejecting the null hypothesis in 100 simulated datasets. [Blue square, univariate gene; Purple circle, univariate metaboltie; Orange triangle, concatenation; Black star, Fisher's method; Red diamond, multivariate extension]

In Figure 3, we increase the set size for the metabolites ($N_{M_{s}}=20$), to make them comparable in size to the gene sets ($N_{G_{s}}=20$), but continue to generate the metabolite intensities with a lower effect size than the gene expression values. Notice the change in the concatenated data results (orange triangles) from Figure 2, where the *N*_*gene *_= 1000 dataset dominated the *N*_*metabolite *_= 200 dataset in the concatenated list. Now, for the Fisher's exact test (panel A) the strength of a single enriched platform is muddied by the non-enriched platform (e.g., sets 13-18). However, the set detection frequency is improved for the 25% enrichment sets (i.e., 19-21) showing rates exceeding either single platform method. For the logistic regression tests (panel B) the concatenated data is still related to the gene expression data due to the higher effect sizes of the gene expression data compared to the metabolite data. The other combined p-values (black star) and 2-df Wald test (red diamond, panel B) appear to be improved for the low enrichment case of 25% enrichment for genes and metabolites (i.e., sets 19-21) showing the utility of joint enrichment methods in marginal cases.

The sum-of-squared statistics test (panel C) continues to perform maximally for all tests. However, these self-contained tests identify all of the random sets (25-29) as enriched. Given that 12% of the genes and metabolites are simulated to be differential in the full dataset these five sets will have 12% enrichment on average. These sets are not detected by the competitive tests (panels A and B) because of their use of relative estimation.

To ensure that our power gains are real we must also look at the error rate of these tests. We consider the Type I error rates across the 41 null sets in 100 simulations. In both scenarios, given in Figures 2 and 3, there are high error rates when the data sets are concatenated and the Fisher's exact test is used (0.297 and 0.408, respectively). Likewise this occurs for the logistic regression model (0.0527 and 0.285, respectively). This error comes from a high rate of depletion calls, that is identification of sets that have fewer significant genes than expected. This problem is greatest when the set size increases. When there are 40 elements per set in the concatenated list, zero differential elements is significantly smaller than the 12% expected by random selection.

The p-values combined by Fisher's method also show inflated error rates for the competitive tests when $N_{M_{s}}=20$; 0.132, 0.0771. This again is a symptom of detecting depleted sets. Recall that in these competitive tests the sample size for the test is based on the number of elements. The larger set size may offer stronger depletion results that are then amplified by the joining of the two tests.

We do not observe error inflation in the sum-of-squares statistic methods. Firstly, the p-value is calculated for a one-sided test. Thus, as currently defined, the sum of squares test cannot detect depletion. Second, the p-values are determined by permutation so there is a limit on the level of precision for the p-values which thus limits the precision of the p-value as combined by summation in the Fisher's method.

### Disjoint set simulation results

Now that we have the general pattern of operating characteristics for each of these methods let us explore some specific hypotheses using the disjoint simulations. Recall that in these simulations we generate data for 50 disjoint sets, of which 10 are designed to be enriched. The correlation structure is homogeneous in that each set has the same structure. However, there is no correlation simulated between sets.

Here we use a different metric to assess the results of the methods. Specifically, we ask, if we were to choose the top ten sets by ranking p-values, would we select the 10 associated sets? Instead of looking at frequencies of being in the top 10 we consider the sum of the ranks for the 10 associated sets. When the 10 associated sets form the top 10 sets selected the sum of the ranks is $R=\sum_{x=1}^{10}x=55$. When there is no association between the set and disease then the 10 sets of interest should have a sum of the ranks with range (55,455) and *E*(*R*) = 255. Boxplots are availble in Additional File 1 for graphical representation of the results presented here.

Under the null model of no enrichment, that is *d*_1 _= *d*_0 _= *c*_1 _= *c*_0 _= 0, the rank sum of the associated sets fall nicely around *E*(*R*) = 255. Under the null model of uniform enrichment, that is *d*_1 _= *d*_0 _= *c*_1 _= *c*_0 _= *δ *we also see that the rank sum of the associated sets matches *E*(*R*) = 255 when *δ *= 0.05 and when *δ *= 0.10. We next assume that on average 25% of the elements in the associated sets are differential, that is *d*_1 _= *c*_1 _= 0.25 and *d*_0 _= *c*_0 _= 0. This results in an overall 5% rate of differential elements within the datasets. The sum-of-squares statistic *R *achieves nearly perfect rank sums for all tests when $N_{M_{s}}=20$ and only falter for the metabolite only tests when $N_{M_{s}}=4$ (mean ± sd: 126.5 ± 38.3). In the competitive tests there is improvement in *R *when any of the joint tests are used compared to the univariate gene or metabolite tests. For example, the 2-degree of freedom test has mean score 85.3 (±24.8 sd, where $N_{M_{s}}=4$) compared to the gene-only or metabolite-only logistic tests with means 101.1 ± 25.6 and 166.7 ± 38.9, respectively. The correlation in this scenario was 0.20 within the sets. If we increase the correlation to 0.60 we find that *R *increases in the competitive tests, specifically the logistic tests become 119.3 ± 25.6 for the joint test, 138.3 ± 28.8 for genes only, and 185.8 ± 42.0 for metabolites only ($N_{M_{s}}=4$). This loss of power is possibly due to loss of information attributed to the dependent measurements. Thankfully such high correlations are not likely to be present in real applications (e.g., [14]). Considering a scenario in which few elements are differential, we reduce the parameters *d*_1 _= *c*_1 _= 0.1 with *d*_0 _= *c*_0 _= 0. Since *d*_1 _and *c*_1 _are probabilities we expect that on average 10% of the elements of the associated sets are differential and we focus only on scenarios where $N_{M_{s}}=20$. The overall enrichment is 2% on average so the competitive tests still perform better than if the sets were randomly assigned. It may be the case that as few as one, or none of the elements are simulated to be differential. Due to these low counts we see an increase in *R *for the sum-of-squared statistics (e.g. mean ± sd: 66.3 ± 16.3 for the 2-df test).

Finally, we consider a scenario with noise in the null sets, that is *d*_1 _= *c*_1 _= 0.25 and *d*_0 _= *c*_0 _= 0.05. It is in this scenario that the sum-of-squared statistic begins to falter (e.g. mean ± sd: 159.8 ± 15.8 for the 2-df test). In fact we see that, beyond an increase in *R*, under this scenario the joint enrichment test performs more poorly than the univariate tests of the gene or metabolites alone (140.2 ± 16.8 and 114.0 ± 18.7, respectively). It is not surprising that this self-contained test performs poorly as this non-specific behavior is a criticism of self-contained method. It is curious, however, that the joint methods appear to fare worse in this situation.

### Application to prostate metabolomics and transcriptomic data

As a final application of the above methods, we utilize the metabolomic data of Sreekumar et al. (2009) [1] and the gene expression data from the same samples (GEO:GSE8511). We consider the comparison between localized tumor (n = 12) and benign tissues (n = 16) and use the Kyoto Encyclopedia of Genes and Genomes (KEGG, version 50, April 2009) to determine the set mapping. Of 518 well measured metabolites, 147 metabolites that can be mapped to the KEGG sets. Of the >40,000 gene probes measured on the Agilent Whole Human Genome microarray, 2169 genes that can be mapped to KEGG. There are 98 KEGG sets in which at least one gene and one metabolite are measured.

**Figure 4:**
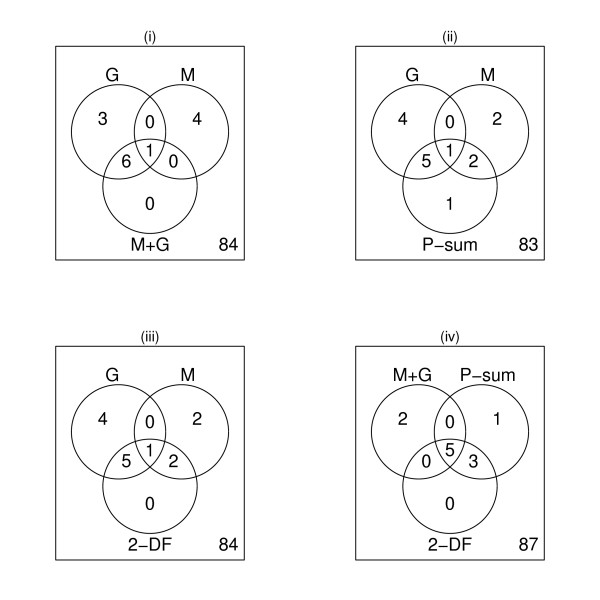
**Venn diagrams comparing enrichment methods for prostate cancer data**. Set enrichment was determined using the logistic regression model. Panels i, ii, and iii compare the sets identified as enriched for genes (G) or metabolites (M) alone to a joint enrichment method; (i) Univariate naïve, (ii) Fisher's method (p-value sum), (iii) 2-df Wald test. Panel iv compares the three joint tests.

Each of the enrichment methods is run on this data. As this is experimental data, we do not know the true association of the genes and metabolites with the KEGG sets. To assess our results we compare the findings of each method. Figure 4 shows a selection of these comparisons using the logistic regression model and a p-value threshold of *p *< 0.05. Here we see that only combining p-values via p-value summation (i.e., Fisher's method; panel ii) detects a set not already detected by one or more of the univariate methods (panels i - iii). However, the joint models provide a more refined list of sets compared to using the union of the results of the two univariate methods. It may be preferable to consider those sets with a significant joint association as preferred candidates for follow-up. Panel (iv) of Figure 4 compares the results of these three joint enrichment methods. We see that in this situation the sum of the p-values by Fisher's method selects nearly the same sets as the 2-df Wald test. Since we are not assuming correlation between *γ *and *μ *the 2-df Wald test is simply a sum of the univariate Wald statistics so its behavior should be similar to the sum of -*log*(*p*_γ_) and -*log*(*p*_*μ*_) as in the Fisher's method.

The Fisher's exact test methods behaved similarly to the logistic regression tests shown in Figure 4. The sum-of-squared statistics tests were overly liberal identifying over 90% of the pathways as enriched. This implies that there was a high rate of differential elements throughout the datasets. Such background noise makes a competitive test the preferred choice of enrichment test. Additionally this may suggest that the KEGG pathway maps, as applied, may not accurately capture the co-regulation in the data.

## Conclusions

In this work we have considered set enrichment testing methods for the joint analysis of transcriptomic and metabolomic datasets. We consider several procedures including two novel methods: the logistic regression 2-degree of freedom Wald test and the 2-dimensional permutation p-value for the sum-of-squared statistics test. Through a novel simulation model and design we explored the properties of these tests in relation to their univariate counterparts. We find that the joint tests can improve our ability to detect results that are marginal univariately, as evidenced in Figures 2 and 3. We also find that joint tests improve the ranking of associated pathways compared to their univariate counterparts.

The various joint methods performed similarly for most simulations. The concatenation of datasets and the Fisher's method of combining p-values had inflated error in the competitive test. For the logistic regression test, the 2-df wald test currently peforms similarly to the Fisher's method for combining the two p-values, due to the assumption that *ρ*_*γμ *_= 0. Non-zero correlation would have provided a weighted sum in the 2-df test. Though we were not estimating *ρ*_*γμ *_to be non-zero, the slightly inflated error rate of the Fisher's method test, suggests that the independence assumption may not always hold and dependent methods should be considered [15]. In future work we will continue to explore if and when correlation may be a contributing factor or if there are other methods for combining the tests in a weighted fashion either at the level of the test statistic or p-value. One of the more attractive features of the 2-df Wald test and 2-dimensional permutation test is that they can easily be extended to n-dimensions. This will allow for the incorporation of multiple omics platforms such as proteomics, genomics, or gene copy number. The data concatenation and sum of p-values methods can also be extended, but this may compound their potential error.

## Authors' contributions

All authors contributed to the study concept and approved the final version of the submitted manuscript. Study design, simulation, and manuscript preparation was contributed by LMP, JMT and DG. Data analysis and graphics preparation were performed by LMP. LMP assumes responsibility as guarantor of the study.

## Supplementary Material

Additional file 1 **Supplementary Information**. The file Poisson_IntegrativeEnrichment_Supp.pdf contains supplementary information about the paper. Click here for file

Additional file 2 **R code**. The file Poisson_IntegrativeEnrichment_SimRCode.zip contains the R code for performing the simulations, along with implementations of the methodology. Click here for file
