# Supplementary material for: Integrative set enrichment testing for multiple omics platforms
Source: BMC Bioinformatics. 2011 Nov 25;12:459. doi: 10.1186/1471-2105-12-459 (PMC3329720; doi:10.1186/1471-2105-12-459)
Supplement: Additional file 1 — Supplementary Information. The file Poisson_IntegrativeEnrichment_Supp.pdf contains supplementary information about the paper. [file 1471-2105-12-459-S1.PDF]

Supplement for: Integrative set enrichment testing for multiple omics platforms

Laila M. Poisson, Jeremy M.G. Taylor, Debashis Ghosh

March 18, 2011

1 Bootstrap Estimation of Correlation

For the joint assessment we begin by modelling the genes and metabolites separately using the absolute value of the per-element t-statistic as the measure of differential ability. We chose to use the absolute t-statistic instead of $-log_{10}(p)$ because it appeared to be more stable in bootstrap resampling described below. Thus for set S we fit the following two models:

$$logit(Pr(G_j \in S)) = \gamma_0 + \gamma(|T_j^G|) \tag{1}$$

$$logit(Pr(M_k \in S)) = \mu_0 + \mu(|T_k^M|). \tag{2}$$

We construct a joint test of $H_{02}^{LR} : \gamma = 0, \mu = 0$ using a two degree-of-freedom Wald test. Specifically, $EV^{-1}E^T$, where $E = [\hat{\gamma}, \hat{\mu}]$, and V is an estimated variance-covariance matrix for γ and μ. We can obtain estimates of the variance of γ and μ from the univariate model estimates. However we do not have a convenient estimate of the correlation between the two parameters.

To estimate the correlation between the esimates $\hat{\gamma}_S$ and $\hat{\mu}_S$, for set S, we construct the bootstrap distributions of the two parameters, say $\underline{\tilde{\gamma}} = (\tilde{\gamma}_1, \tilde{\gamma}_2, \ldots, \tilde{\gamma}_B)$ and $\underline{\tilde{\mu}} = (\tilde{\mu}_1, \tilde{\mu}_2, \ldots, \tilde{\mu}_B)$. We use row-resampling which reflects the use of genes and metabolites, not subjects, as input for the model. Through simulation we found that subject-resampling, which does not reflect the use of genes and metabolites as input for the logistic regression model, dramatically underestimates the variance of the parameters compared to the variance estimates obtained from the univariate mod-

els of Equations 1 and 2. Additionally, when resampling the elements, we stratify the sample by inclusion in S to retain a fixed number of elements in S in each bootstrap sample. That is, for the set S we can split $\underline{T}^G$ into $\underline{T}^{GS}$ and $\underline{T}^{GS'}$. We sample $n(S_G)$ genes from $\underline{T}^{GS}$ with replacement, where $n(S_G)$ is the number of genes in S. The remainder of the genes are sampled with replacement from $\underline{T}^{GS'}$. The metabolite test statistics are then resampled in the same fashion. Stratification is especially important for platforms that tend to have small set counts.

Upon generation of B bootstrap estimates of γ and μ, $\underline{\tilde{\gamma}} = (\tilde{\gamma}_1, \tilde{\gamma}_2, \ldots, \tilde{\gamma}_B)$ and $\underline{\tilde{\mu}} = (\tilde{\mu}_1, \tilde{\mu}_2, \ldots, \tilde{\mu}_B)$, we can compute an estimate of the correlation $\rho_{\gamma\mu}$ =corr$(\underline{\tilde{\gamma}}, \underline{\tilde{\mu}})$. To reduce convergence error associated with small samples sizes and logistic regression bootstrapping we use a one-step bootstrap procedure as described by Moulton and Zeger (1991) Moulton and Zeger [1991]. Essentially the iterated weighted least-squares (IWLS) estimation algorithm is seeded with the observed parameters values of $(\hat{\gamma}_0, \hat{\gamma})$ for the gene expression. Then, given the bth bootstrap resampled vector $\underline{T}^{Gb}$, one step is taken in the IWLS algorithm and the new estimate $(\tilde{\gamma_0}^b, \tilde{\gamma}^b)$ is reported. The IWLS algorithm is stopped here and not allowed to continue to convergence. One-step estimation is particularly important for models on small sets where the full IWLS can have problems of separation resulting in estimates nearing positive or negative infinity. Such non-convergent estimates inflate the variance and in simulation the variance estimates tended to be large compared to their model based counterparts with some variances on the order of 10^3 and greater when $n(S) = 4$.

Through simulation we find that there is not strong correlation between the parameters $\hat{\gamma}$ and $\hat{\mu}$ even in sets where the genes and metabolites were simulated to be correlated. Figure 1 shows a histogram of N correlation estimates, each from $B = 500$ bootstrap resamples, for a set S in which there is correlation between genes, $\rho_{GG} = 0.6$, between metabolties, $\rho_{MM} = 0.6$, and between genes and metabolites, $\rho_{MG} = 0.25$. The correlation in S' is not homogeneous and most genes and metabolites are simulated to be independent.

We see in Figure 1, i that the distribution of the estimates are fairly symmetric about zero. If we perform Fisher's transformation on the correlations to arrive at standardized z-scores we see that the estimates are underdispersed compared to the quantiles of a standard normal distribution; see Figure 1, ii. The loss of correlation is likely due to the row-resampling that is used for this bootstrap.

Given these findings, we assume that the correlation is zero between $\hat{\gamma}$, and $\hat{\mu}$ for all sets. Thus V

i ii

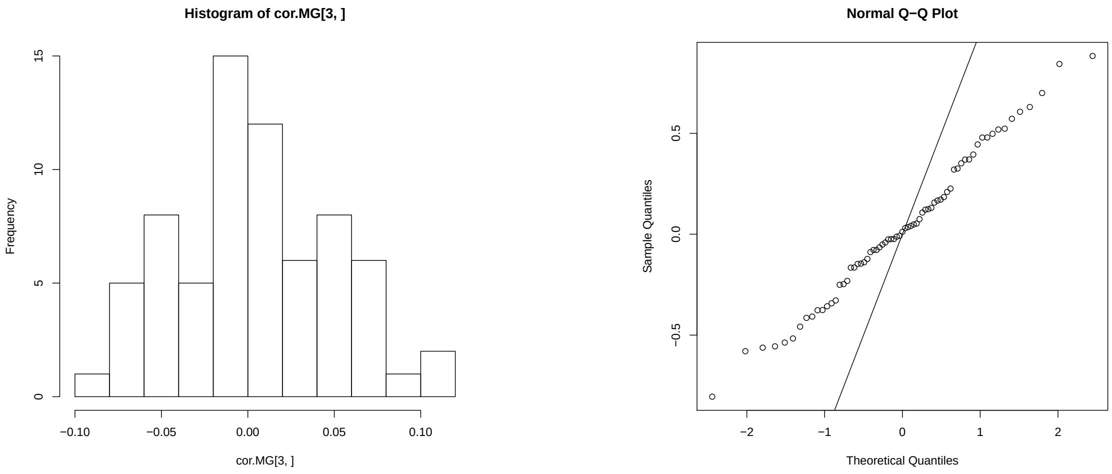

Figure 1: **Correlation of γ and μ** (i) Correlation of the estimates of γ and μ do not differ much from zero. (ii) They are under-dispersed compared to the expected normal distribution under the Fisher's z-score transformation.

can be estimated as a diagonal vector with $var(\hat{\gamma})$ and $var(\hat{\mu})$ estimated from the univarite models. This reduces our test statistic to the sum of the two one-degree-of-freedom tests. Specifically, the test statistic for set S can be written as $U_S^{LR} = EV^{-1}E^T = \hat{\gamma}^2\sigma_\gamma^{-2} + \hat{\mu}^2\sigma_\mu^{-2}$, where $E = [\hat{\gamma}, \hat{\mu}]$, and $V = diag(\sigma_\gamma^2, \sigma_\mu^2)$. We assume that $U_S^{LR} \sim \chi_2^2$ under the null hypothesis $H_{02}^{LR} : \gamma = 0, \mu = 0$.

2 Example of set indicator matrix

For clarity, an example of the indicator matrix, m_{ks}, for the inclusion of metabolite k in set s, is given in Table 1. This matrix represents a dataset of 200 metabolites assigned to 70 sets of size $N_{M_k} = 4$. Notice that accoring to Table 2, 100% of the metabolites of set $s = 1$ are drawn from distribution $h = 1$. Likewise 50% of the metabolites in set $s = 10$ are drawn from distritubion $h = 1$ and 50% are drawn from $h = 0$. The randomly drawn sets $s = 25$ and $s = 29$ are shown. Notice that set $s = 25$ does not include any of the differential metabolites, $k \in (1, \ldots, 36)$, where as one differential metabolite, $k = 4$, was selected to be included in set $s = 29$. Sets $s \in (30, \ldots, 70)$ are formed by a partitioning of the null metabolites, $h = 0$ and $k \in (37, \ldots, 200)$.

k	h	1	2	⋯	10	11	⋯	25	⋯	29	30	⋯	45	46	47	⋯	70
1	1	1	0	⋯	1	0	⋯	0	⋯	0	0	⋯	0	0	0	⋯	0
2	1	1	0	⋯	0	0	⋯	0	⋯	0	0	⋯	0	0	0	⋯	0
3	1	1	0	⋯	0	0	⋯	0	⋯	1	0	⋯	0	0	0	⋯	0
4	1	1	0	⋯	1	0	⋯	0	⋯	0	0	⋯	0	0	0	⋯	0
5	2	0	1	⋯	0	1	⋯	0	⋯	0	0	⋯	0	0	0	⋯	0
6	2	0	1	⋯	0	0	⋯	0	⋯	0	0	⋯	0	0	0	⋯	0
7	2	0	1	⋯	0	1	⋯	0	⋯	0	0	⋯	0	0	0	⋯	0
8	2	0	1	⋯	0	0	⋯	0	⋯	0	0	⋯	0	0	0	⋯	0
⋮	⋮	⋮	⋮	⋱	⋮	⋮	⋱	⋮	⋱	⋮	⋮	⋱	⋮	⋮	⋮	⋱	⋮
36	9	0	0	⋯	0	0	⋯	0	⋯	0	0	⋯	0	0	0	⋯	0
37	0	0	0	⋯	0	0	⋯	1	⋯	0	1	⋯	0	0	0	⋯	0
38	0	0	0	⋯	0	0	⋯	0	⋯	0	1	⋯	0	0	0	⋯	0
39	0	0	0	⋯	0	0	⋯	0	⋯	0	1	⋯	0	0	0	⋯	0
40	0	0	0	⋯	0	0	⋯	0	⋯	1	1	⋯	0	0	0	⋯	0
⋮	⋮	⋮	⋮	⋱	⋮	⋮	⋱	⋮	⋱	⋮	⋮	⋱	⋮	⋮	⋮	⋱	⋮
99	0	0	0	⋯	0	0	⋯	1	⋯	0	0	⋯	1	0	0	⋯	0
100	0	0	0	⋯	0	0	⋯	1	⋯	0	0	⋯	1	0	0	⋯	0
101	0	0	0	⋯	0	0	⋯	0	⋯	0	0	⋯	0	1	0	⋯	0
102	0	0	0	⋯	1	0	⋯	0	⋯	0	0	⋯	0	1	0	⋯	0
103	0	0	0	⋯	0	0	⋯	0	⋯	0	0	⋯	0	1	0	⋯	0
104	0	0	0	⋯	0	0	⋯	0	⋯	1	0	⋯	0	1	0	⋯	0
105	0	0	0	⋯	0	0	⋯	0	⋯	0	0	⋯	0	0	1	⋯	0
106	0	0	0	⋯	0	0	⋯	0	⋯	0	0	⋯	0	0	1	⋯	0
107	0	0	0	⋯	0	0	⋯	0	⋯	0	0	⋯	0	0	1	⋯	0
108	0	0	0	⋯	0	1	⋯	0	⋯	0	0	⋯	0	0	1	⋯	0
⋮	⋮	⋮	⋮	⋱	⋮	⋮	⋱	⋮	⋱	⋮	⋮	⋱	⋮	⋮	⋮	⋱	⋮
152	0	0	0	⋯	1	0	⋯	1	⋯	0	0	⋯	0	0	0	⋯	0
153	0	0	0	⋯	0	0	⋯	0	⋯	1	0	⋯	0	0	0	⋯	0
⋮	⋮	⋮	⋮	⋱	⋮	⋮	⋱	⋮	⋱	⋮	⋮	⋱	⋮	⋮	⋮	⋱	⋮
200	0	0	0	⋯	0	1	⋯	0	⋯	0	0	⋯	0	0	0	⋯	1
N_{M_k}		4	4	⋯	4	4	⋯	4	⋯	4	4	⋯	4	4	4	⋯	4

Table 1: **This matrix represents an example m_{ks} matrix** for 200 metabolites (rows) and 70 sets (columns) with set size $N_{M_k} = 4$. The metabolite number, k, and the distribution from which it was drawn, $h \in (0, 1, \ldots, 9)$, are listed to the left of the indicator matrix.

Table 2: **Structure of sets 1–24.** Each set S contains N_{G_s} genes and N_{M_s} metabolites drawn such that π-percent of the elements are from a differential distribution $h \in (1, 2 \ldots, 9)$ and the remainder are from the null distribution $h = 0$.Sets 25–29 are constructed by random draws across all 10 distributions, $h \in (0, 1, \ldots, 9)$. Sets $30 - 70$ are a disjoint partition of the null set, $h = 0$, so that each element in this set contributes to at least one set.

π	h	1	2	3	4	5	6	7	8	9
1		1	2	3	4	5	6	7	8	9
0.5		10	11	12	13	14	15	16	17	18
0.25		19	20	21	22	23	24	-	-	-

3 Heterogeneous set simulation results

Following are some results from the heterogeneous set simulation. For each simulation scenario, 100 datasets were generated and tested. In Figure 2 we depict the frequency with each set, from $1 - 29$, was determined to be significant at $\alpha = 0.05$ for each test considered. The average rate of false positives is computed across the 41 null sets per test. Boxplots of these error rates across the 100 simulated data sets are presented in Figure 3. That is, one point on the boxplot represents the average error rate for that test in one simulated dataset.

4 Disjoint set simulation results

Let us explore some specific hypotheses using the disjoint simulations. Recall that in these simulations we generate data for 50 disjoint sets, of which 10 are designed to be enriched. The correlation structure is homogeneous in that each set has the same structure. However, there is no correlation simulated between sets.

Here we use a different metric to assess the results of the methods. Specifically, we ask, if we were to choose the top ten sets by ranking p-values, would we select the 10 associated sets? Instead of looking at frequencies of being in the top 10 we consider the sum of the ranks for the 10 associated sets. When the 10 associated sets form the top 10 sets selected the sum of the ranks is $R = \sum_{x=1}^{10} x = 55$. When there is no association between the set and disease then the 10 sets of interest should have a sum of the ranks with range $(55, 455)$ and $E(R) = 255$. Boxplots are available in the online supplement for graphical representation of the results presented here.

Under the null model of no enrichment, that is $d_1 = d_0 = c_1 = c_0 = 0$, the rank sum of the

associated sets fall nicely around $E(R) = 255$; see Figure 6. Under the null model of uniform enrichment, that is $d_1 = d_0 = c_1 = c_0 = \delta$ we also see that the rank sum of the associated sets matches $E(R) = 255$ when $\delta = 0.05$ as in Figure 7 and when $\delta = 0.10$.

We next assume that on average 25% of the elements in the associated sets are differential, that is $d_1 = c_1 = 0.25$ and $d_0 = c_0 = 0$. This results in a 5% rate of differential elements within the datasets. Plots are show for $N_m = 4$ (figure 8) and for $N_m = 20$ (figure 9).

Under the higher correlation model shown in Figures 10 and 11, R is larger in the competitive tests compared to the lower correlation model of Figures 8 and 9. The loss of power is possibly due to loss of information attributed to the dependent measurements.

We next consider the behavior of the test in a scenario of few differential elements; see Figure 12. Here we set $d_1 = c_1 = 0.1$ and $d_0 = c_0 = 0$. The overall enrichment is 2% on average so the competitive tests still perform better than if the sets were randomly assigned. Since d_1 and c_1 are probabilities we expect that on average 10% of the elements of the associated sets are differential. It may be the case that one, or none of the elements are simulated to be differential. These low counts likely contribute to the increase in R for the sum-of-squared statistics in Figure 12.

Finally, we consider the model where $d_1 = c_1 = 0.25$ but $d_0 = c_0 = 0.05$, that is we simulate noise in the null sets; see figure 13. It is in this scenario that the sum-of-squared statistic begins to falter. In fact we see that, beyond an increase in R, under this scenario the joint enrichment test performs more poorly than the univariate tests of the gene or metabolites alone. It is not surprising that this self-contained test performs poorly as this non-specific behavior is a criticism of self-contained method. It is surprising, however, that the joint methods appear to fare worse in this situation. However, notice that R is still less than $E(R) = 255$. Each of the tests falters when the noise and signal rates are close, see figure 14. Here $d_1 = c_1 = 0.10$ and $d_0 = c_0 = 0.05$ simulating a scenario where the association of molecular change with set is low.

References

L.H. Moulton and S.L. Zeger. Bootstrapping generalized linear models. *Computational Statistics and Data Analysis*, 11:53–63, 1991.

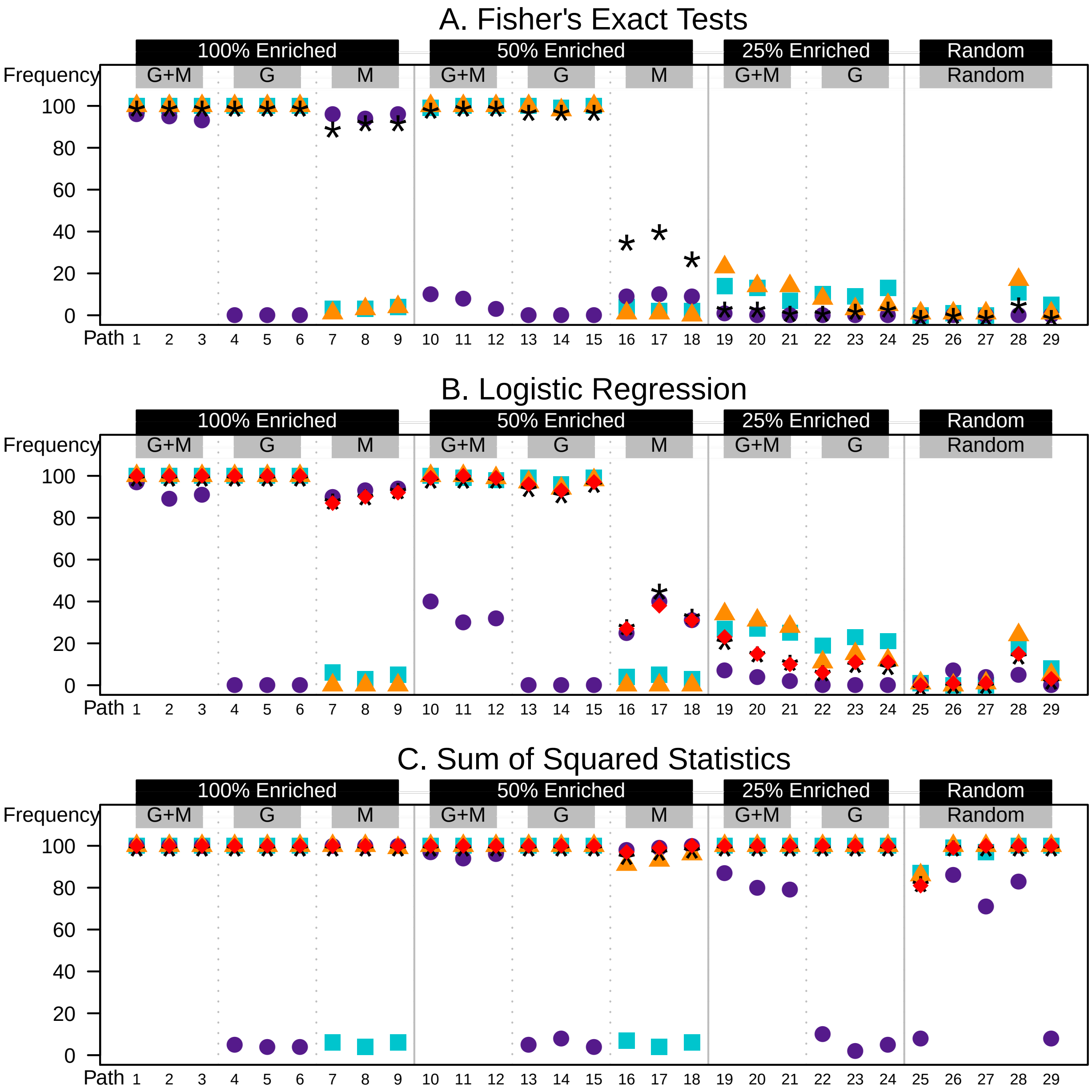

Figure 2: **1000 genes and 200 metabolites generated for 30 samples** Four metabolites and 20 genes are included in each set with $\rho_{GG} = \rho_{MM} = 0.20$, and $\rho_{MG} = 0.10$. The symbols represent the frequency of rejecting the null hypothesis in 100 simulated datasets. [Blue square, univariate gene; Purple circle, univariate metaboltie; Orange triangle, concatenation; Black star, Fisher's method; Red diamond, multivariate extension]

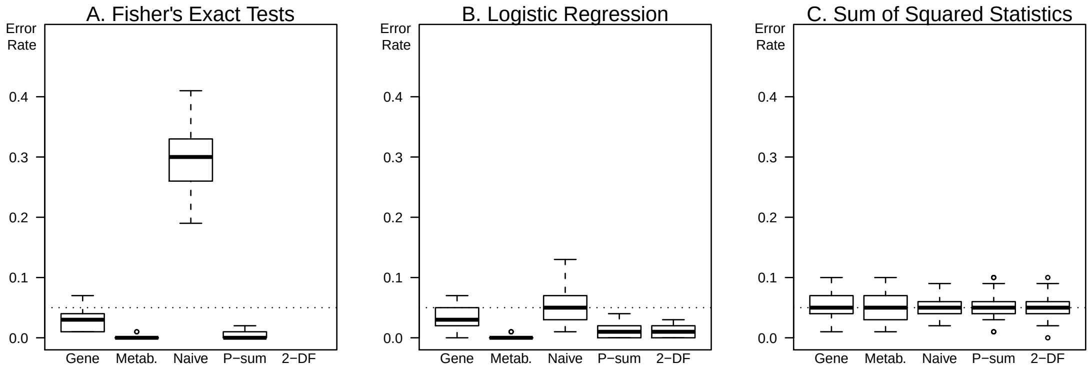

Figure 3: **Type I error associated with Figure 2.** Each boxplot represents 100 measurements of the error rate across the 41 null sets.

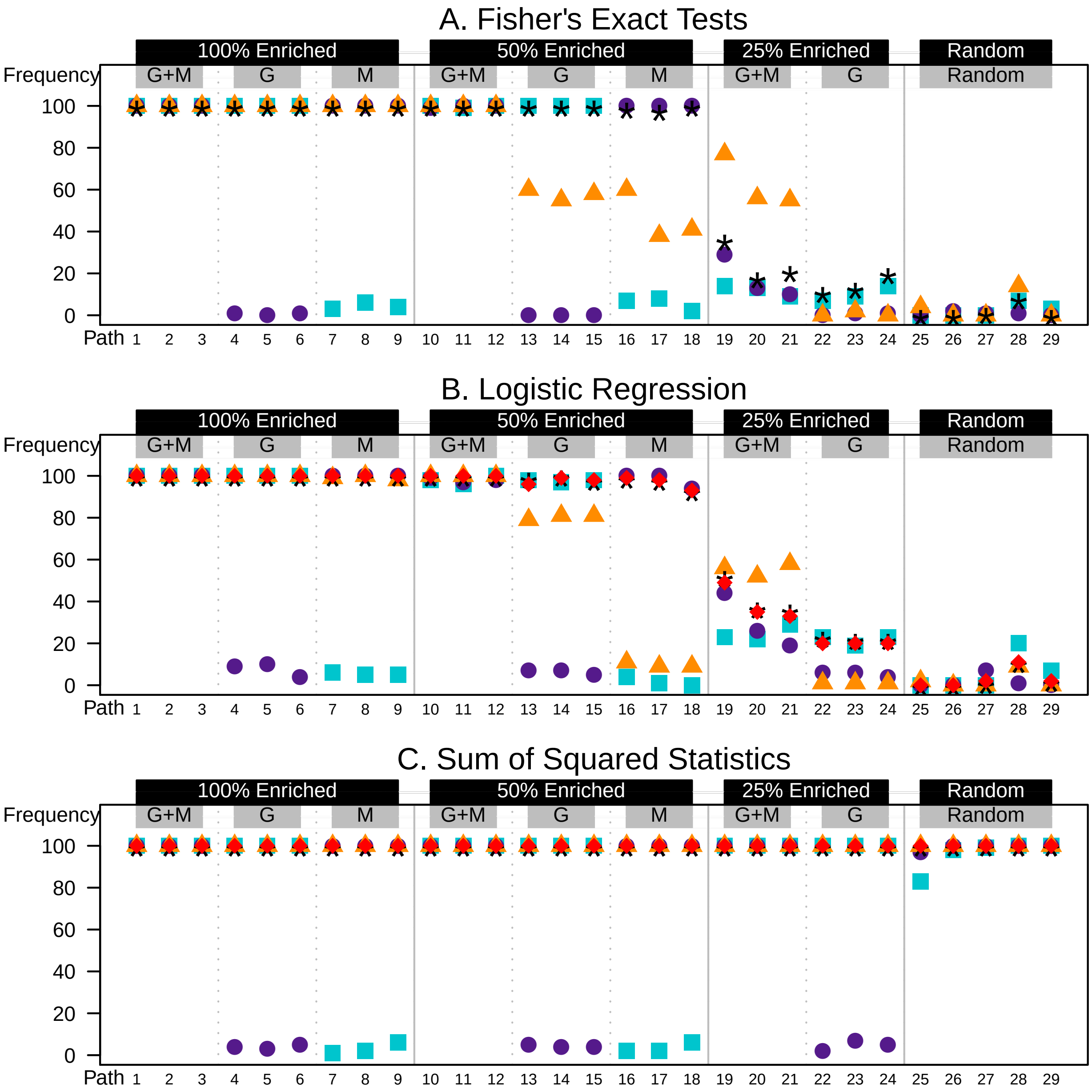

Figure 4: **1000 genes and 1000 metabolites generated for 30 samples** Each set includes 20 metabolites and 20 genes with $\rho_{GG} = \rho_{MM} = 0.20$, and $\rho_{MG} = 0.10$. The symbols represent the frequency of rejecting the null hypothesis in 100 simulated datasets. [Blue square, univariate gene; Purple circle, univariate metaboltie; Orange triangle, concatenation; Black star, Fisher's method; Red diamond, multivariate extension]

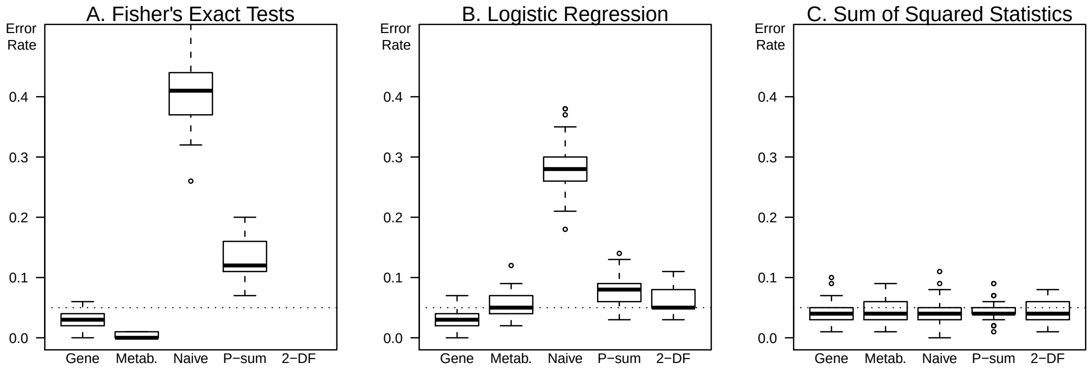

Figure 5: **Type I error associated with Figure 4.** Each boxplot represents 100 measurements of the error rate across the 41 null sets.

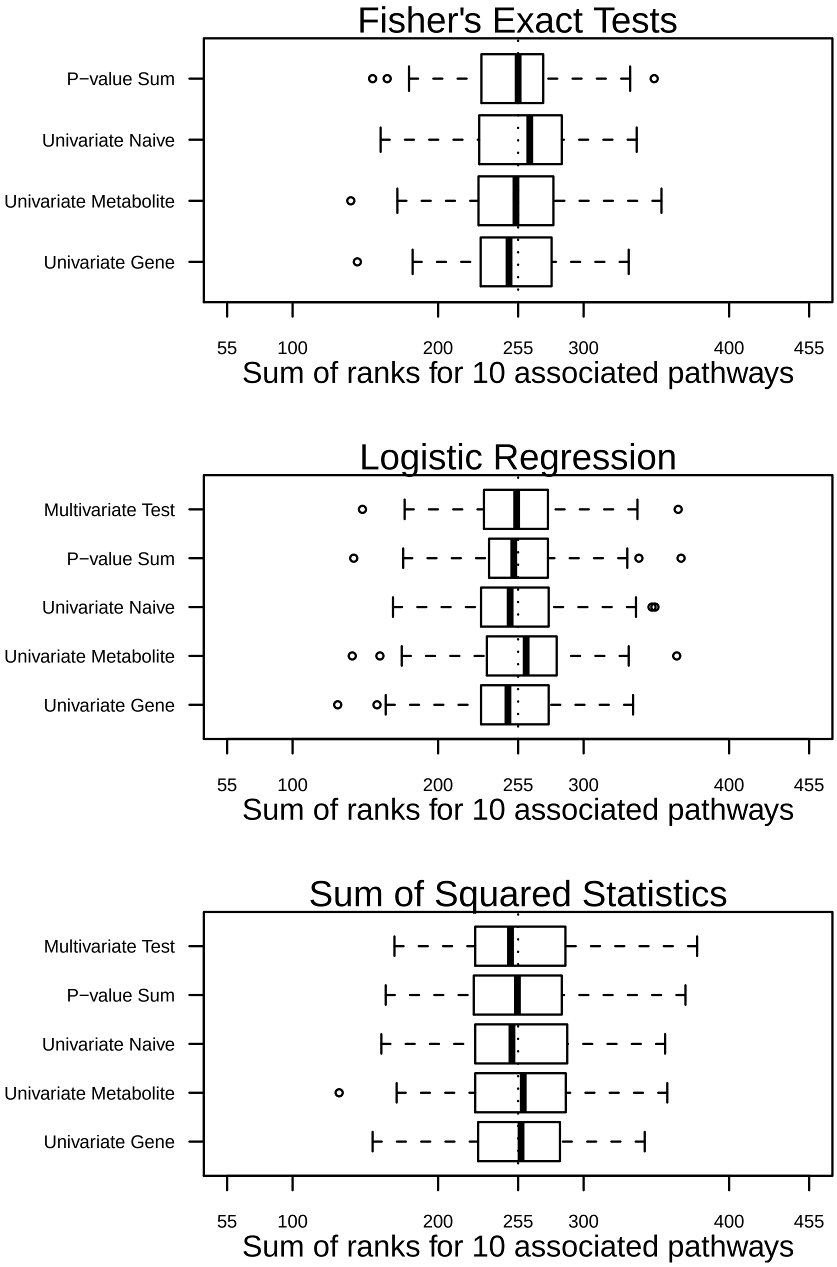

Figure 6: **The sum of the ranks of the 10 associated sets assuming no differential elements** were measured, that is $d_1 = d_0 = c_1 = c_0 = 0$. The correlation structure $\rho_{GG} = \rho_{MM} = 0.20$, $\rho_{MG} = 0.1$ is assumed. Each of the 50 disjoint set were simulated to contain 20 metabolites and 20 genes. $N_{sample} = 30$.

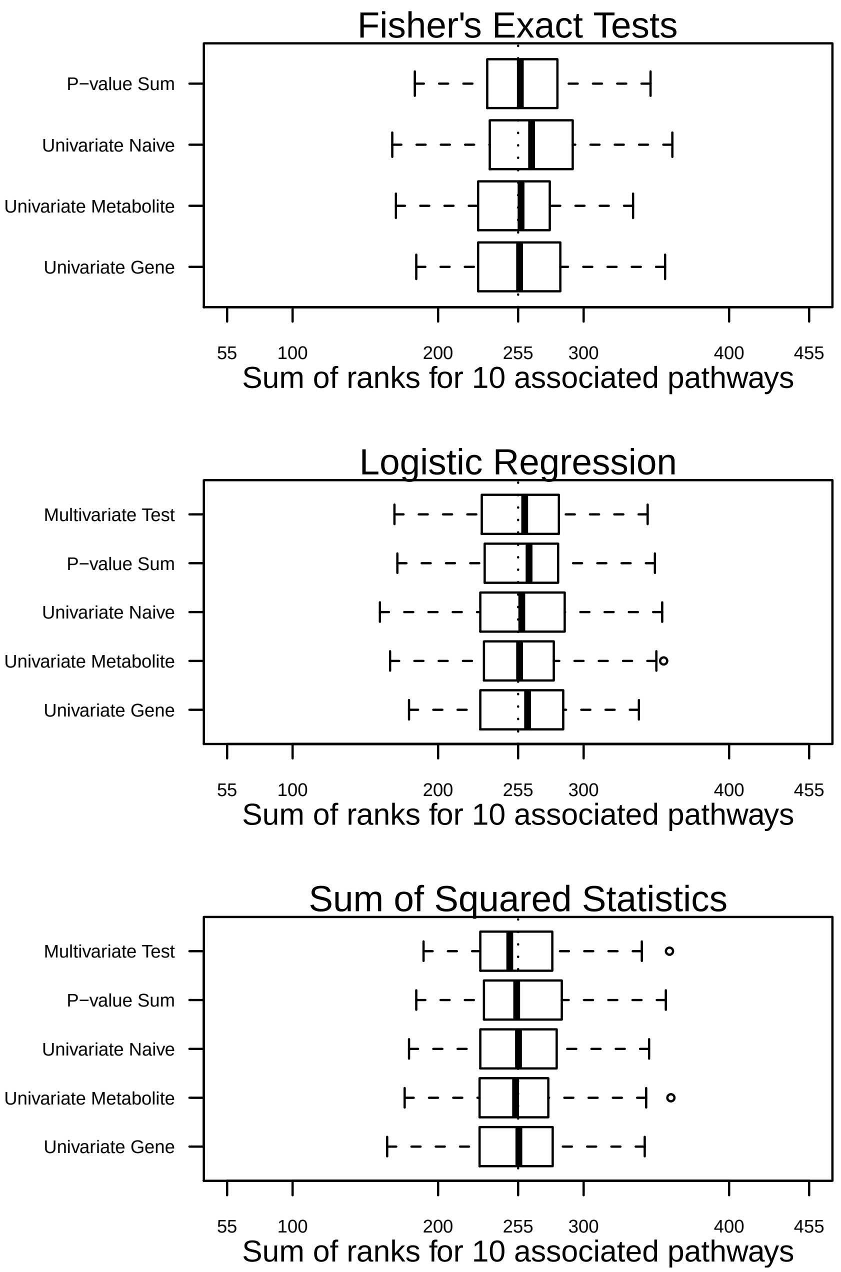

Figure 7: **The sum of the ranks of the 10 associated sets assuming a constant probability of differential elements** across the sets measured, that is $d_1 = d_0 = c_1 = c_0 = 0.05$. The correlation structure $\rho_{GG} = \rho_{MM} = 0.20$, $\rho_{MG} = 0.1$ is assumed. Each of the 50 disjoint set were simulated to contain 20 metabolites and 20 genes. $N_{sample} = 30$.

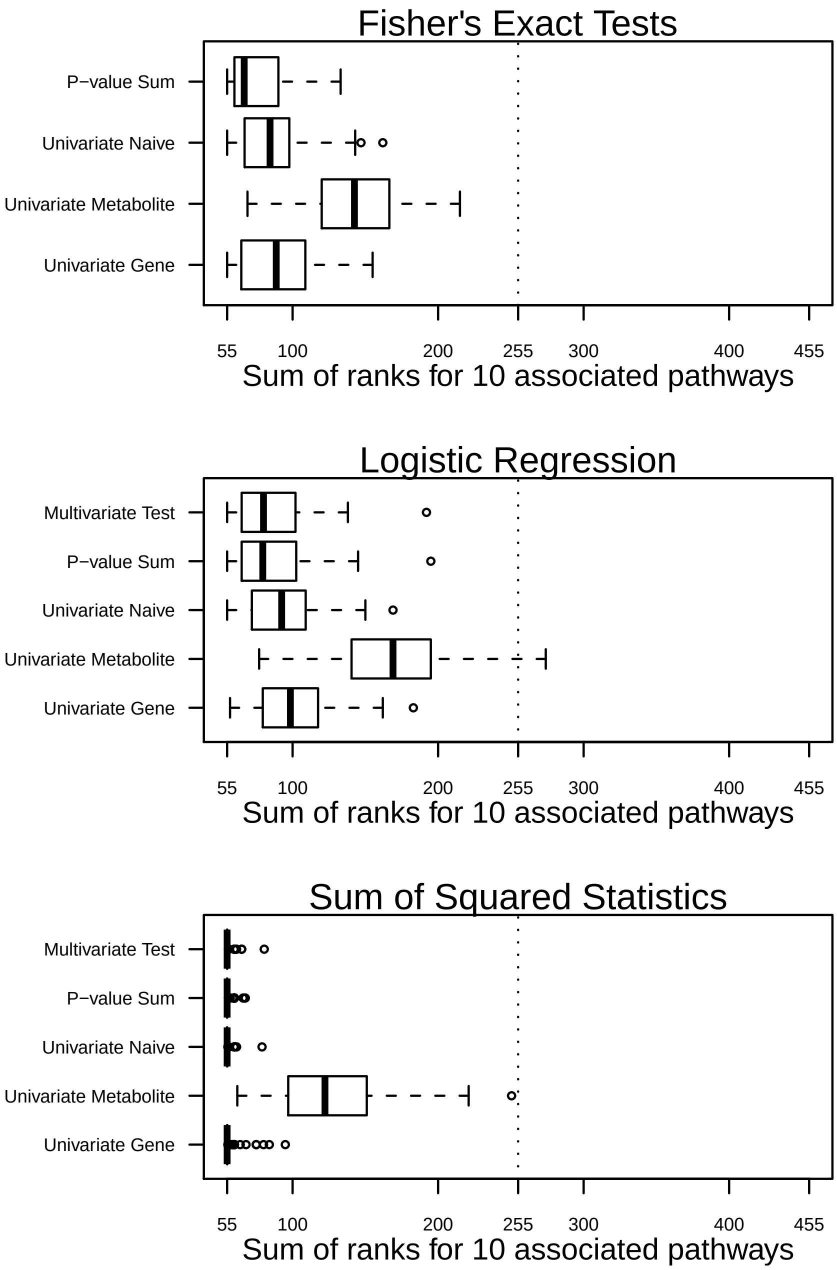

Figure 8: **The sum of the ranks of the 10 associated sets assuming 25% of the elements are differential** in these sets, that is $d_1 = c_1 = 0.25$ and $d_0 = c_0 = 0$. The correlation structure $\rho_{GG} = \rho_{MM} = 0.20$, $\rho_{MG} = 0.1$ is assumed. Each of the 50 disjoint set were simulated to contain 4 metabolites and 20 genes. $N_{sample} = 30$.

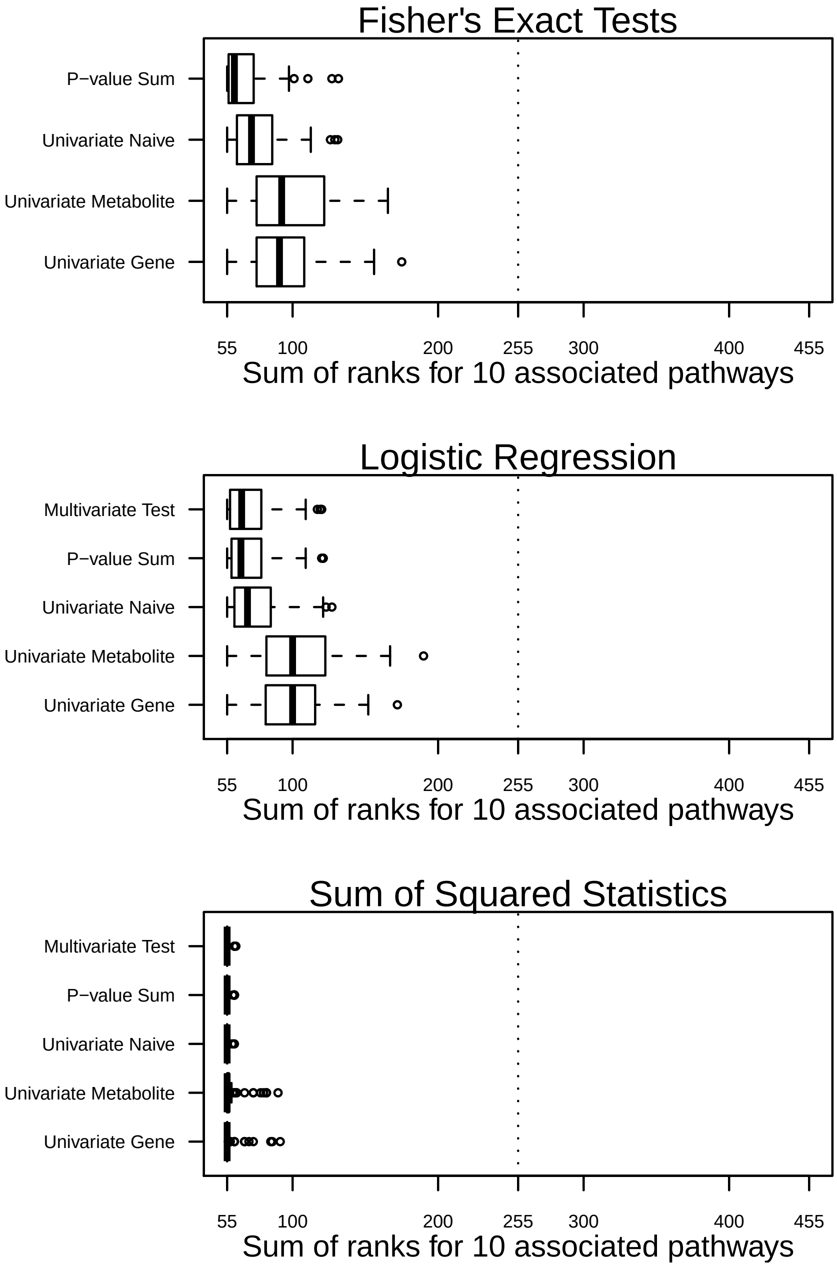

Figure 9: **The sum of the ranks of the 10 associated sets assuming 25% of the elements are differential** in these sets, that is $d_1 = c_1 = 0.25$ and $d_0 = c_0 = 0$. The correlation structure $\rho_{GG} = \rho_{MM} = 0.20$, $\rho_{MG} = 0.1$ is assumed. Each of the 50 disjoint set were simulated to contain 20 metabolites and 20 genes. $N_{sample} = 30$.

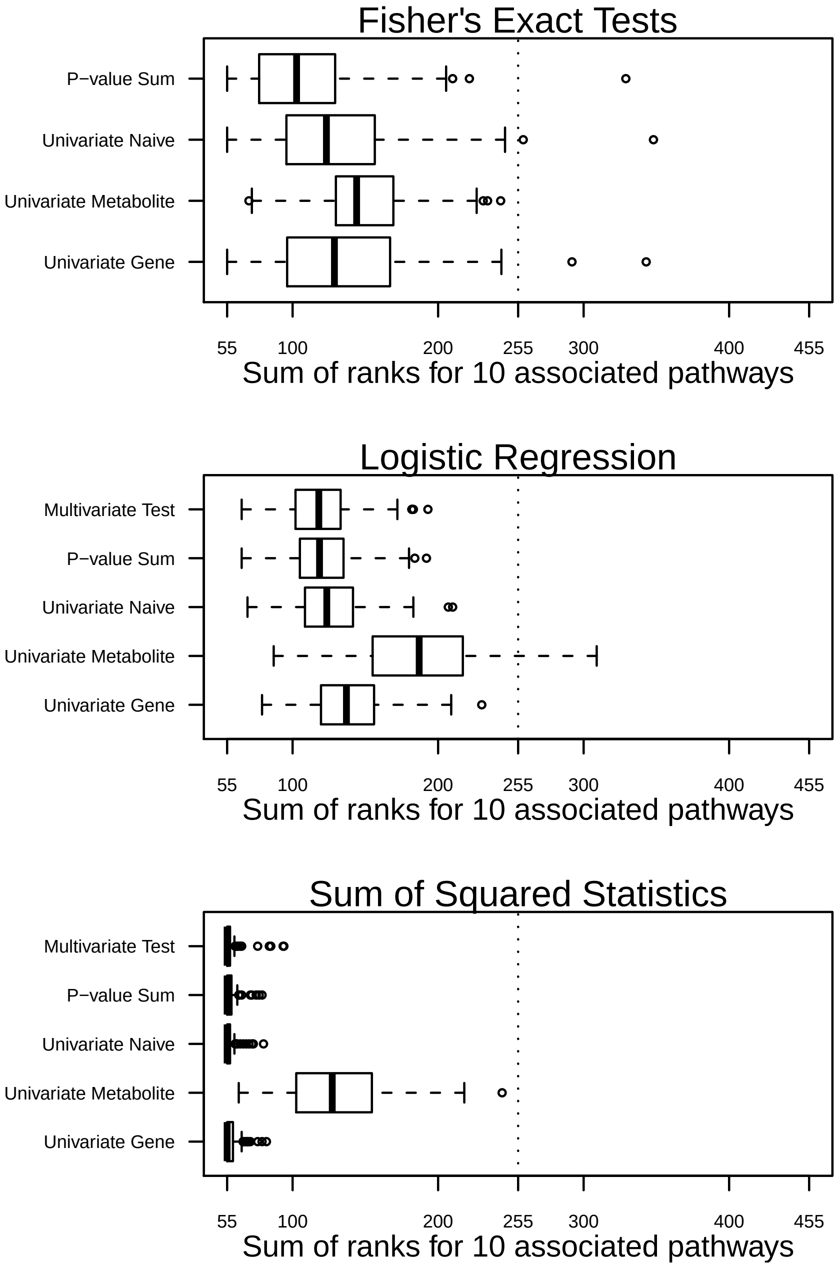

Figure 10: **The sum of the ranks of the 10 associated sets assuming 25% of the elements are differential** in these sets, that is $d_1 = c_1 = 0.25$ and $d_0 = c_0 = 0$. The correlation structure $\rho_{GG} = \rho_{MM} = 0.60$, $\rho_{MG} = 0.25$ is assumed. Each of the 50 disjoint set were simulated to contain 4 metabolites and 20 genes. $N_{sample=30}$.

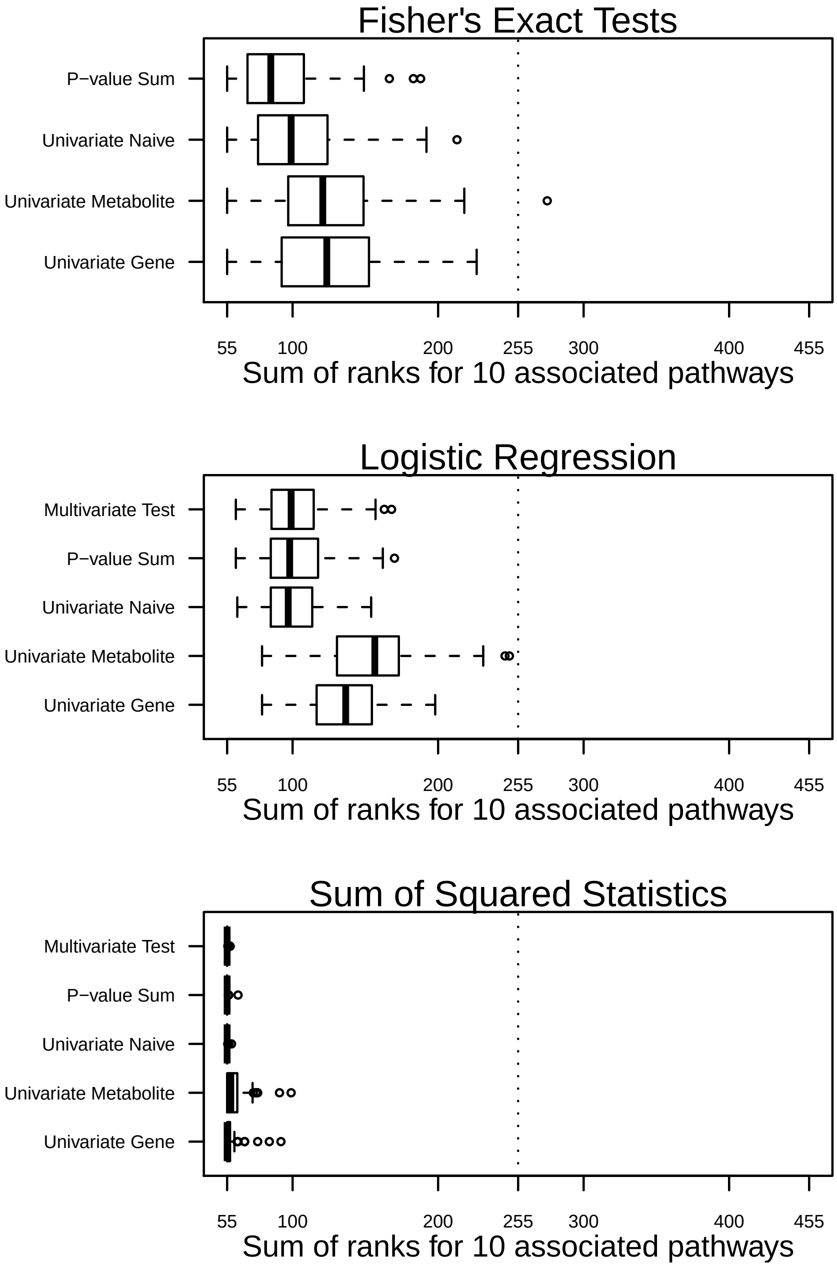

Figure 11: **The sum of the ranks of the 10 associated sets assuming 25% of the elements are differential** in these sets, that is $d_1 = c_1 = 0.25$ and $d_0 = c_0 = 0$. The correlation structure $\rho_{GG} = \rho_{MM} = 0.60$, $\rho_{MG} = 0.25$ is assumed. Each of the 50 disjoint set were simulated to contain 20 metabolites and 20 genes. $N_{sample=30}$.

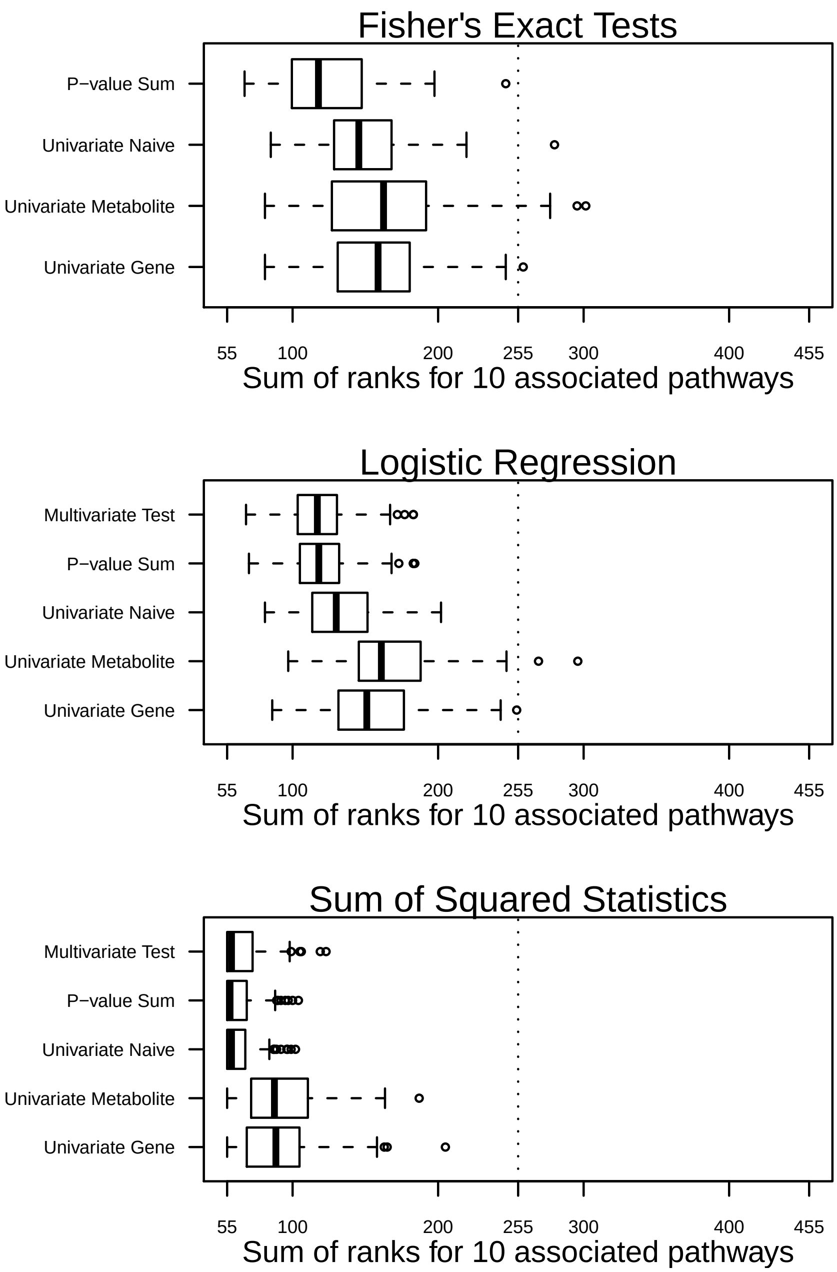

Figure 12: **The sum of the ranks of the 10 associated sets assuming 10% of the elements are differential** in these sets, that is $d_1 = c_1 = 0.10$ and $d_0 = c_0 = 0$. The correlation structure $\rho_{GG} = \rho_{MM} = 0.20$, $\rho_{MG} = 0.1$ is assumed. Each of the 50 disjoint set were simulated to contain 20 metabolites and 20 genes. $N_{sample} = 30$.

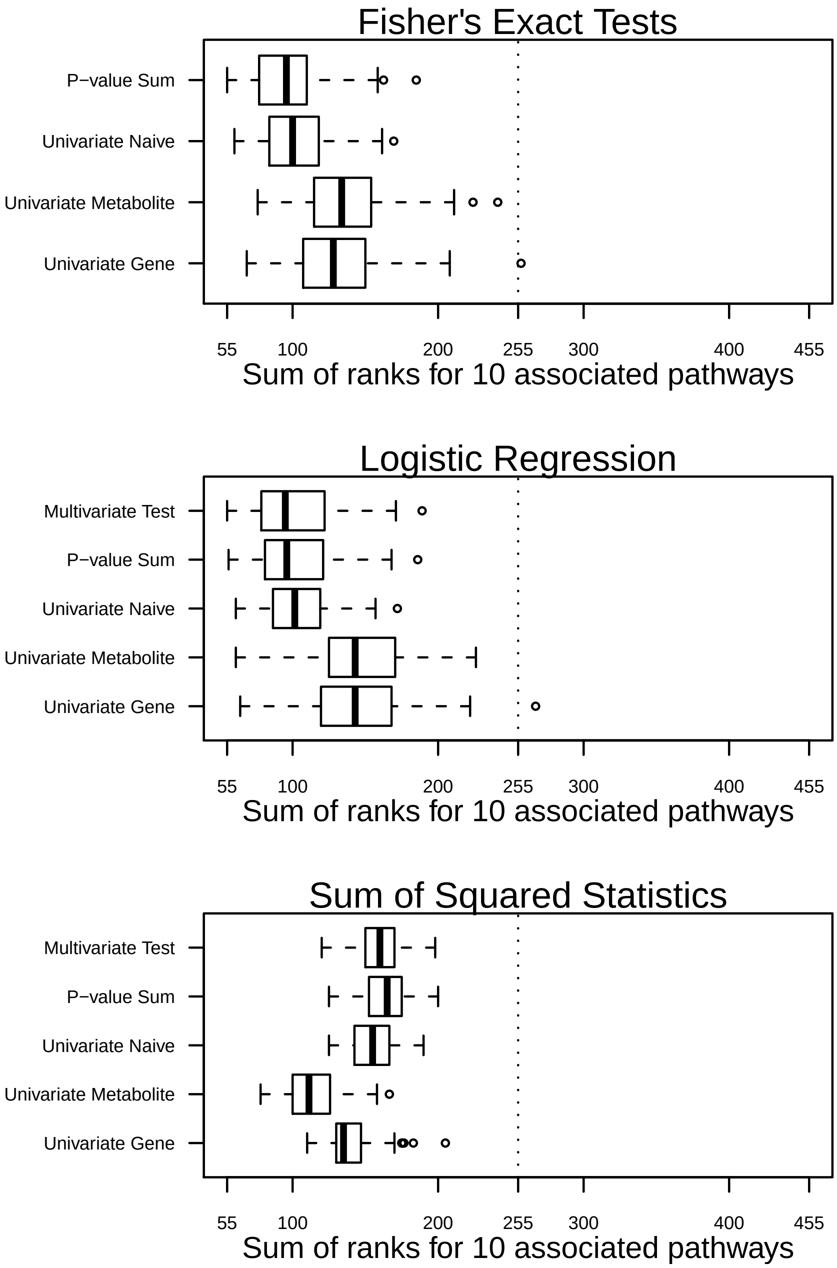

Figure 13: **The sum of the ranks of the 10 associated sets assuming 25% of the elements are differential** in these sets, that is $d_1 = c_1 = 0.25$ and 5% of the elements are differential in the remaining sets, $d_0 = c_0 = 0.05$. The correlation structure $\rho_{GG} = \rho_{MM} = 0.20$, $\rho_{MG} = 0.1$ is assumed. Each of the 50 disjoint set were simulated to contain 20 metabolites and 20 genes. $N_{sample} = 30$.

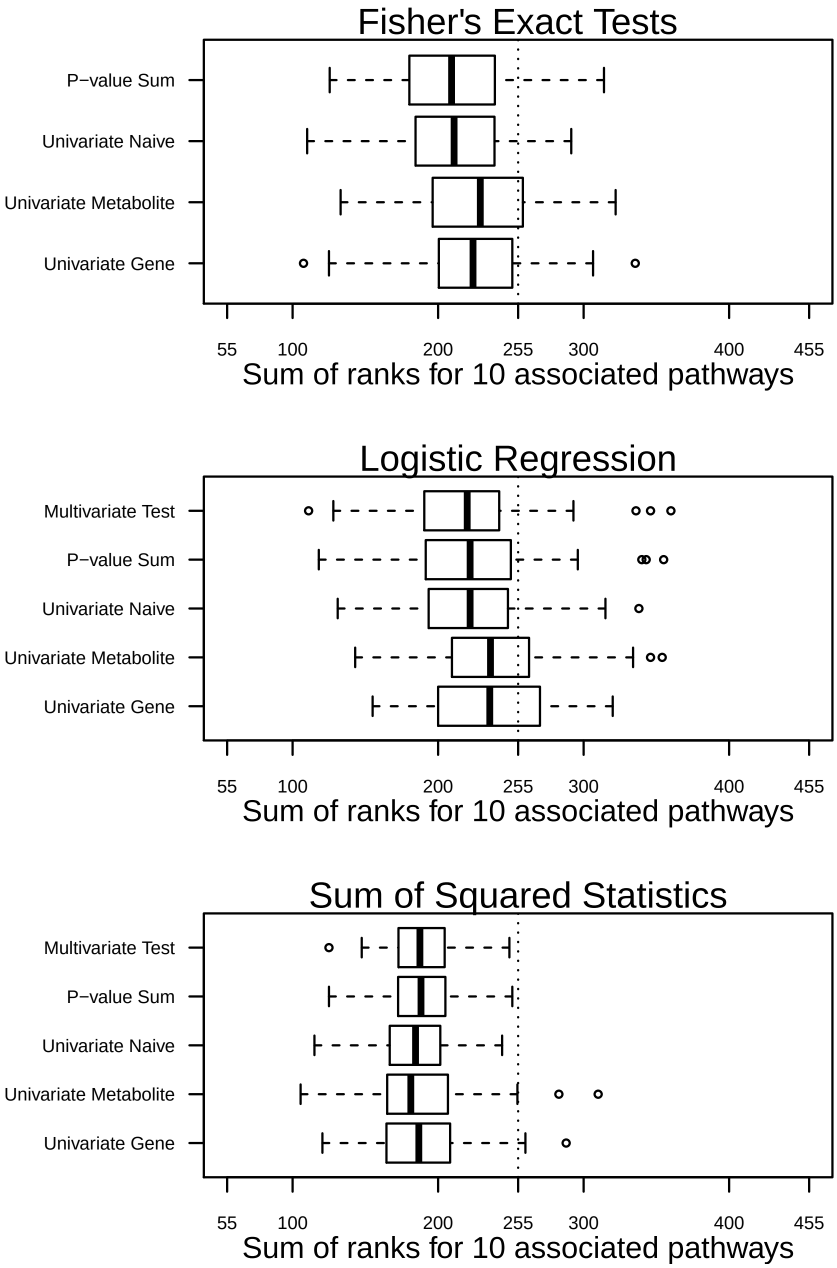

Figure 14: **The sum of the ranks of the 10 associated sets assuming 25% of the elements are differential** in these sets, that is $d_1 = c_1 = 0.10$ and 5% of the elements are differential in the remaining sets, $d_0 = c_0 = 0.05$. The correlation structure $\rho_{GG} = \rho_{MM} = 0.20$, $\rho_{MG} = 0.1$ is assumed. Each of the 50 disjoint set were simulated to contain 20 metabolites and 20 genes. $N_{sample} = 30$.
